# A Targeted and Protease-Activated Genetically Encoded Melittin-Containing Particle for the Treatment of Cutaneous and Visceral Leishmaniasis

**DOI:** 10.1021/acsami.4c10426

**Published:** 2024-09-06

**Authors:** Madiha Habib, Jiale Zheng, Chin-Fung Chan, Zaofeng Yang, Iris L. K. Wong, Larry M. C. Chow, Marianne M. Lee, Michael K. Chan

**Affiliations:** † School of Life Sciences and Center of Novel Biomaterials, The Chinese University of Hong Kong, Shatin, Hong Kong SAR 999077, China; ‡ Department of Applied Biology and Chemical Technology and the State Key Laboratory of Chemical Biology and Drug Discovery, The Hong Kong Polytechnic University, Hung Hom, Hong Kong SAR 999077, China

**Keywords:** Cry3Aa crystal protein, melittin, antileishmanial peptides, *Leishmania*, parasites

## Abstract

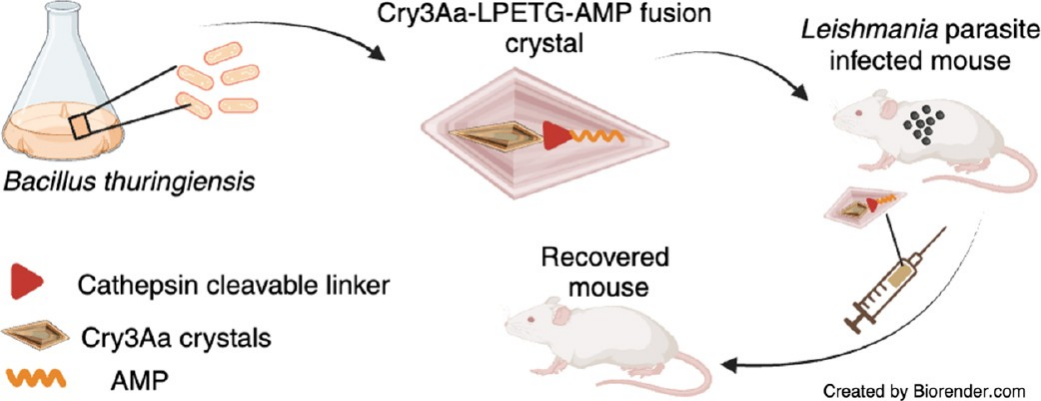

Intracellular infections are difficult to treat, as pathogens can take advantage of intracellular hiding, evade the immune system, and persist and multiply in host cells. One such intracellular parasite, *Leishmania*, is the causative agent of leishmaniasis, a neglected tropical disease (NTD), which disproportionately affects the world’s most economically disadvantaged. Existing treatments have relied mostly on chemotherapeutic compounds that are becoming increasingly ineffective due to drug resistance, while the development of new therapeutics has been challenging due to the variety of clinical manifestations caused by different *Leishmania* species. The antimicrobial peptide melittin has been shown to be effective in vitro against a broad spectrum of *Leishmania*, including species that cause the most common form, cutaneous leishmaniasis, and the most deadly, visceral leishmaniasis. However, melittin’s high hemolytic and cytotoxic activity toward host cells has limited its potential for clinical translation. Herein, we report a design strategy for producing a melittin-containing antileishmanial agent that not only enhances melittin’s leishmanicidal potency but also abrogates its hemolytic and cytotoxic activity. This therapeutic construct can be directly produced in bacteria, significantly reducing its production cost critical for a NTD therapeutic. The designed melittin-containing fusion crystal incorporates a bioresponsive cathepsin linker that enables it to specifically release melittin in the phagolysosome of infected macrophages. Significantly, this targeted approach has been demonstrated to be efficacious in treating macrophages infected with *L. amazonensis* and *L. donovani* in cell-based models and in the corresponding cutaneous and visceral mouse models.

## 1 Introduction

Leishmaniasis is a protozoan disease caused by *Leishmania* parasites that is transmitted by sandflies.^1^ It is estimated that more than 12 million people are infected worldwide, with 1.3 million new cases added every year.^2^ The disease is endemic, predominantly in the tropical and subtropical regions, and disproportionately affects the poorest segments. Notably, leishmaniasis has been classified as a neglected tropical disease (NTD) and is among one of the disease groups targeted for elimination within the health goal (3.3) of the Sustainable Development Goals created in 2015 aiming to promote an equitable world, including health care equality.^3^

Among the three primary forms of leishmaniasis, namely, cutaneous, mucocutaneous, and visceral, cutaneous leishmaniasis (CL), which produces ulcerative lesions leaving the patients with lifelong scarring, is the most common, while visceral leishmaniasis (VL) which affects the liver and spleen is the most fatal, with a near 100% case-fatality rate if left untreated.^4,5^ Treatment remains challenging, and other options are limited, relying mostly on chemotherapeutic compounds, such as pentavalent antimonials: pentamidine,^6,7^ miltefosine,^8^ aminosidine,^9^ and amphotericin B,^10^ each of which has their own drawbacks, including high price, toxicity, lengthy treatment period and complicated administration, and most pressingly, the emergence of drug resistance.^11,12^

Melittin, a 26-amino acid antimicrobial peptide (AMP) from the honeybee *Apis mellifera* venom, has been shown to be potent against different *Leishmania* species, including *Leishmania donovani, Leishmania major*, and *Leishmania panamensis*.^13−15^ However, its high hemolytic activity and cytotoxicity toward host cells limits its clinical translation and application.^16^ Thus, different approaches have been explored to modulate its toxicity, including modification to the amino acid sequence,^17^ and the encapsulation of melittin in nanostructured delivery vehicles such as liposomes and hydrogels,^18,19^ which not only can sequester melittin from interacting with the surrounding cells but also overcome the challenge of proteolytic susceptibility often associated with peptide-based drugs. Nevertheless, most encapsulation strategies involve multiple steps, including preparation of the encapsulating agents, synthesis of the peptide, and entrapment of the peptide into the encapsulated agent, which contributes to significant production costs as well as inactivation risks of the therapeutic. Given that treatment affordability is one of the primary considerations in the development of a therapeutic for NTDs, a system that can consolidate the entire encapsulation process into one simple step with minimal impact to the peptide would be economically and operationally desirable. Better yet, this system can be endogenously produced cheaply by a workhorse bacterium that is easily adapted for scale-up production.

Our group has developed a unique platform for the delivery of peptides and proteins based on Cry3Aa protein crystals that are naturally synthesized in the bacterium *Bacillus thuringiensis* (*Bt*). Cry3Aa protein has been extensively studied and utilized as a biopesticide for decades due to its insecticidal properties and it being nontoxic to humans.^20,21^ Given Cry3Aa’s in cellulo crystal-forming, biocompatible, and nontoxic properties, we hypothesized that the resultant crystal could be developed as a novel scaffolding platform for the cellular delivery of therapeutic peptides and proteins. Further impetus was provided by the structural analysis of the *in vivo* Cry3Aa crystals showing large solvent channels (∼5 nm wide), which could promote the entrapment of protein/peptide within the particles.^22^ We subsequently demonstrated that fusion of cargo protein/peptides did not abrogate the crystal-forming ability of Cry3Aa protein,^23^ and ultimately used this platform to mediate the efficacious delivery of the antimicrobial peptides, dermaseptin S1 (DS1) and LL-37, to treat leishmaniasis^24^ and *H. pylori* infection,^25^ respectively, as well as myoglobin to enhance radiation treatment against lung cancer.^26^

In our previous study targeting *Leishmania*, we capitalized on the preferential uptake of Cry3Aa crystal by macrophages to facilitate the targeted delivery of DS1 peptide to the *Leishmania*-infected macrophages and demonstrated the efficacious eradication of *L. amazonensis* LV78 in a CL mouse footpad model.^24^ Here, the Cry3Aa-mediated delivery of DS1 was enabled by the high binding affinity of the cationic DS1 peptide to the negatively charged patches within the Cry3Aa crystal solvent channel, which allowed for high AMP loading efficiency. In addition, the entrapped AMP was protected from proteolysis due to the cargo protection conferred by the Cry3Aa crystals.^24^

Although the DS1-bound Cry3Aa crystals (Cry3Aa-DS1) exhibited efficacy toward the induced CL in the infected footpad, they were less effective in the in vivo treatment of *L. donovani*-induced VL—despite a potent IC_50_ value of 0.67 μM for the Cry3Aa-DS1 against *L. donovani* amastigotes compared with >20 μM for free DS1 peptide in the in vitro assay.^24^ Since the therapeutic crystals were directly administered to the footpad lesion by intralesion injection in the CL model, while intravenous administration was used in the VL disease model, we hypothesized that the lack of meaningful response in the latter case could be due to the premature release of the bound peptide from the Cry3Aa crystal before reaching the disease site. Given that we have consistently shown in our other studies that fusion of another protein to Cry3Aa did not disrupt its crystal-forming ability, and significantly, the fused protein retained its function and activity,^23^ we surmised that fusing the AMP to Cry3Aa would be a viable strategy to circumvent the premature release issue. The challenge then was to identify a release system that could be endogenously synthesized and, more importantly, triggered by a stimulus that could promote facile and site-specific cleavage and release of the entrapped AMP to enable high local AMP concentration and minimal systematic toxicity, critical criteria for the highly hemolytic melittin. We thus sought a protease-based endogenous stimulus that would be highly specific to the infected macrophages to minimize the impact on the uninfected macrophages.

Previous studies had reported the upregulation of the lysosomal aspartic proteases, cathepsin D and E, in infected macrophages.^27^ In the study by Antoine and colleagues, the authors showed that the parasitophorous vacuoles in the *Leishmania*-infected macrophages were already enriched with lysosomal proteases, including cathepsin D at the early stage of infection, and that the infected macrophage’s cathepsin D appeared to be membrane bound and predominately distributed on the periphery of the vacuoles while those of the uninfected macrophages were mainly located in the perinuclear vesicles.^28^ We therefore hypothesized that we could exploit these differences in the expression levels and spatial localization of cathepsin D for the trigger and release of melittin in parasite-infected macrophages.

**Scheme 1:**
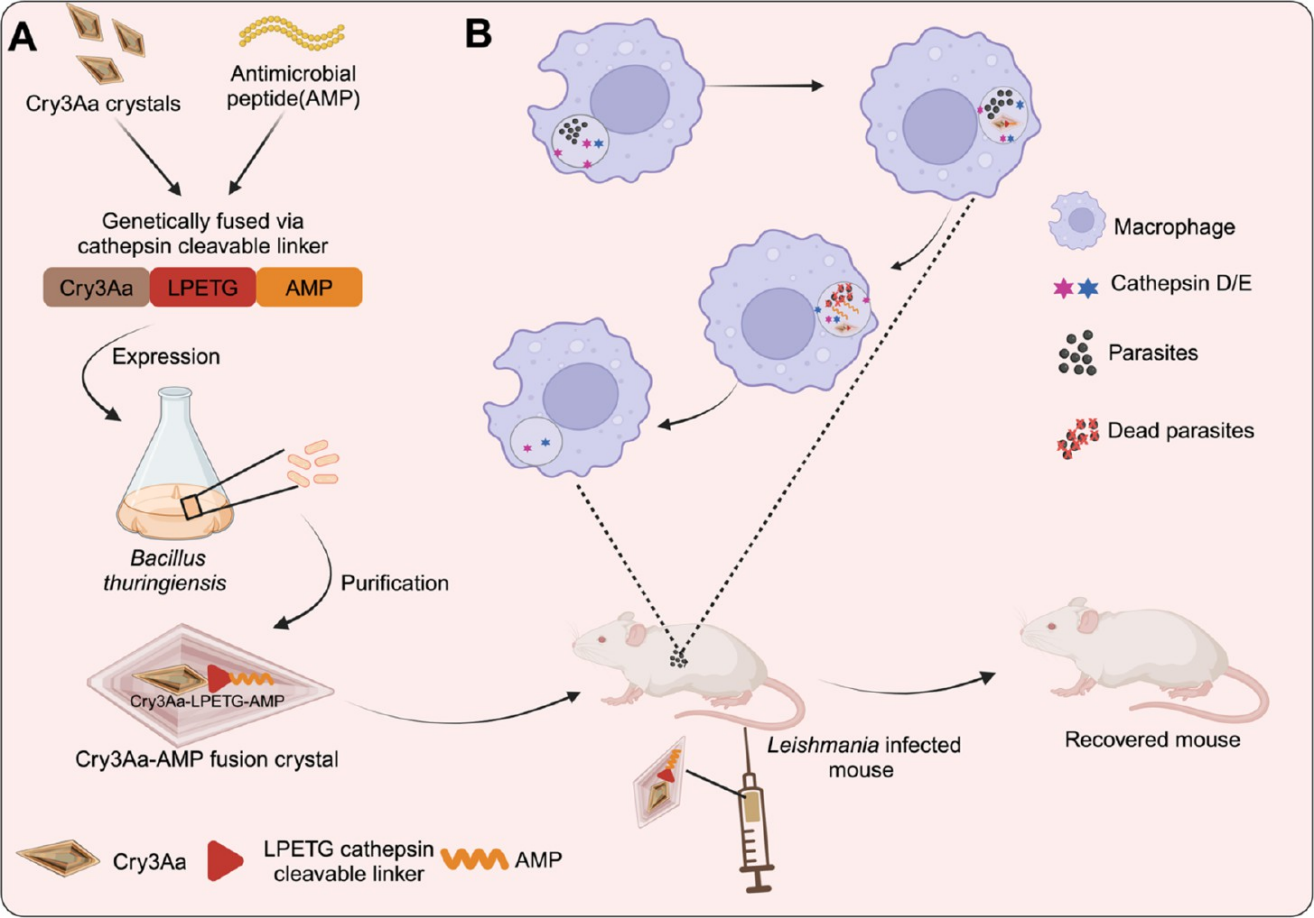
Schematic Illustration of Cry3Aa-AMP Fusion Crystals and Their Use in Treating Leishmaniasis Created with Biorender.com (A) The design and production of Cry3Aa-AMP fusion crystals. (B) Leishmania-infected mouse injected with Cry3Aa-AMP fusion crystals. Preferential uptake of the Cry3Aa-AMP fusion crystals by infected macrophages and the stimuli-responsive release of the AMP from the Cry3Aa therapeutic particle mediated by cathepsin D/E enzymes whose presence is elevated in the acidic environment of infected macrophages.

Herein, we report the modular design and generation of a protein-based antileishmanial agent for the targeted delivery of melittin to *Leishmania*-infected macrophages (Scheme 1). This particle is composed of the Cry3Aa protein that serves as the crystalline scaffold for the genetic fusion of the ensuing molecules: the repurposed sortase recognition sequence as a bioresponsive cathepsin-cleavable linker and the AMP, melittin. We show that the resultant melittin-containing fusion crystals possess not only enhanced stability but also significantly reduced hemolytic activity and cytotoxicity toward erythrocytes and macrophage cells, respectively. Above all, unlike traditional chemotherapeutic drugs, *Leishmania* promastigotes are less prone to acquire resistance against melittin peptide. Notably, these therapeutic crystals are effective against both *L. amazonensis* and *L. donovani* and can elicit an efficacious response in both CL and VL mouse models, suggesting its suitability and potential development as a universal antileishmanial agent against a wide range of *Leishmania* species.

## 2 Results and Discussion

### 2.1 Antimicrobial Resistance Development in *Leishmania* promastigotes

**Figure 1:**
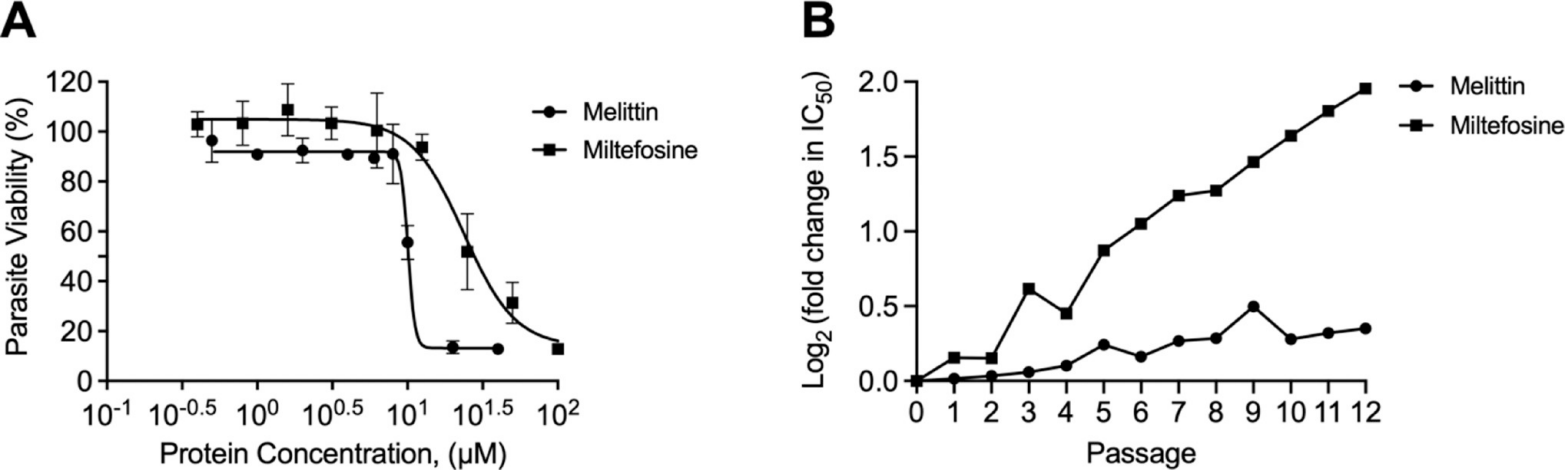
Antileishmanial activity of melittin and miltefosine. (A) Antipromastigote activity of melittin peptide and miltefosine drug against *L. amazonensis* LV78 at different concentrations. (B) Development of resistance in *L. amazonensis* LV78 against melittin peptide and miltefosine. Values represent the fold change in IC_50_ (log_2_) from the starting IC_50_ at passage 0.

Previous studies have reported the antileishmanial effectiveness of melittin toward different *Leishmania* species, including *L. donovani*.^15^ However, to the best of our knowledge, there is no report on its effectiveness on *L. amazonensis*—a major *Leishmania* species that causes CL. Hence, melittin was tested against *L. amazonensis* LV78 promastigotes to evaluate its antileishmanial activity. Miltefosine, the first-line chemotherapeutic drug for treating leishmaniasis, was set up in parallel to serve as a positive control. The resultant IC_50_ of ∼10 μM demonstrated the effectiveness of melittin toward *L. amazonensis* (Figure 1A)—though this value is higher than the IC_50_ of 2.5 μM for *L. donovani* (Figure S1).

Since one of the major advantages touted for AMPs is their reduced susceptibility to antimicrobial resistance, *L. amazonensis* LV78 was treated with either melittin or miltefosine over multiple generations for the evaluation of the relative susceptibility of the parasite to each therapeutic over time. Notably, over the course of 12 generations, the *Leishmania* parasites developed significant resistance to miltefosine but remained susceptible to melittin (Figure 1B). These data support the key benefit of using melittin as an active agent for treating leishmanial diseases.

### 2.2 Design and Production of Fusion Crystals of Cry3Aa-Melittin and Variants

One of the major challenges of using melittin as a therapeutic is its toxicity to mammalian cells.^13,29^ However, previous studies have shown that the encapsulation of melittin in a particle or hydrogel significantly reduced its hemolytic and cytotoxic activities in cells.^30,31^ In a related way, Cry3Aa crystals have been shown to provide protection of fused cargo to proteolytic degradation. We thus hypothesized that the framework of Cry3Aa crystals could likewise be used to sequester melittin and abrogate its toxicity to host cells prior to reaching its target site within macrophages.

Our strategy involved fusing melittin to Cry3Aa equipped with a stimuli-responsive release mechanism to produce a fusion crystal that would keep melittin encapsulated until its delivery to the infected macrophage. As we would like to biosynthesize the antileishmanial fusion crystals in *Bt* cells, the expression of the bactericidal peptide was a concern. Hence, in addition to wild-type melittin, we also explored the fusion of promelittin, the inactive precursor of melittin, to ascertain the impact of the constituent peptide on the expression level of the corresponding Cry3Aa-melittin (Cry3Aa-MLT) and Cry3Aa-promelittin (Cry3Aa-PMLT) fusion crystals.

**Figure 2:**
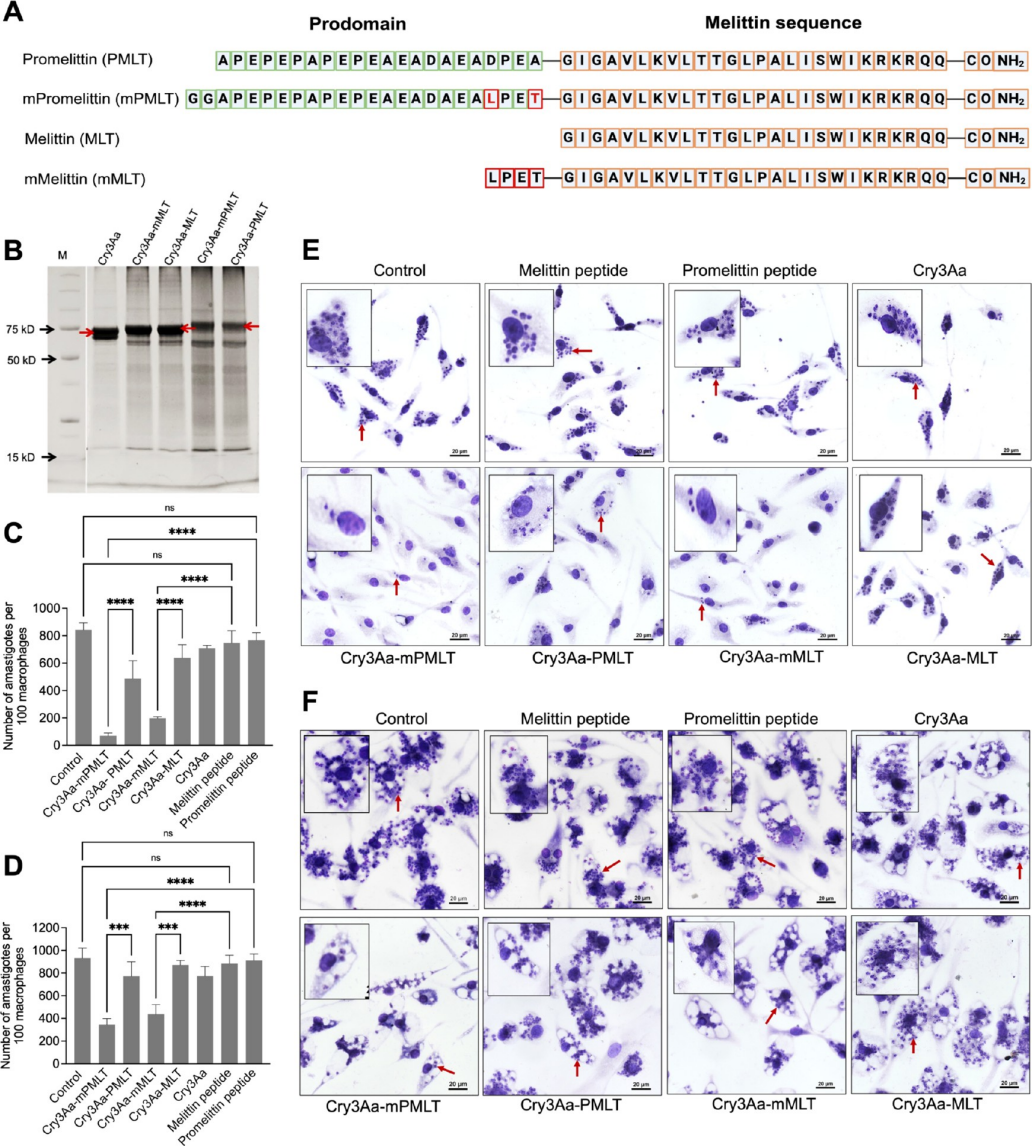
Construction of Cry3Aa-AMP fusion crystals and their antileishmanial activity. (A) Amino acid sequences of promelittin, melittin peptide, and its variants used in the construction of the Cry3Aa-AMP fusion crystals. (B) SDS-PAGE analysis of Cry3Aa-AMP fusion crystals. Cry3Aa-mPMLT, Cry3Aa-mMLT, Cry3Aa-MLT, and Cry3Aa-PMLT were successfully expressed in *Bt* cells and purified by sucrose gradient centrifugation. The molecular weight of Cry3Aa is ∼73 kDa, Cry3Aa-MLT and Cry3Aa-mMLT are ∼75 kDa, and Cry3Aa-PMLT and Cry3Aa-mPMLT are ∼78 kDa. M = molecular weight marker. The bands in each lane slightly greater than 15 kDa are residual lysozyme added during purification. (C–F) Cry3Aa-mediated delivery of melittin to *Leishmania* parasite-infected peritoneal elicited macrophages (PEMs). Quantification of (C) *L. donovani* LU3 amastigotes and (D) *L. amazonensis* LV78 amastigotes in at least 100 randomly selected PEMs after treatment with 0.8 μM of Cry3Aa-AMP fusion crystals or Cry3Aa crystals or free AMP peptides for 72 h. Representative light microscope images of (E) *L. donovani* LU3-infected PEMs and (F) *L. amazonensis* LV78-infected PEMs. The cells were fixed and stained with Giemsa for parasite enumeration. The control groups were untreated parasite-infected-PEMs. Red arrows indicate the presence of parasites in macrophages. ****P < 0.0001 and ***P < 0.001. ns, not significant.

As a means to enhance the release of melittin from the Cry3Aa fusion crystal at the target site, in this case, the phagolysosome of macrophages where the parasites reside, we adopted the design of an LPETG-containing promelittin peptide variant (mPMLT) used in our other studies to generate the Cry3Aa fusion variants of the promelittin (Cry3Aa-mPMLT) and melittin (Cry3Aa-mMLT) (Figure 2A). Although the LPETG sequence is generally recognized as a sortase cleavage motif, computational analysis of the LPETG-containing promelittin sequence by Procleave suggested that the LPETG sequence could potentially aid in enhancing the preferential cleavage at the pro region of melittin by aspartic proteases, including cathepsins D and E (Figure S2). Previous studies had reported that the expression of cathepsin D and E was significantly elevated in stimulated macrophages.^27^ Thus, we hypothesized that LPETG could be employed as a site-specific protease-activated linker to facilitate the release of melittin from the Cry3Aa fusion crystals in phagolysosomes. We therefore engineered the LPETG site between Cry3Aa and the antimicrobial peptide (AMP) to produce Cry3Aa-mPMLT and Cry3Aa-mMLT fusion crystals.

The four Cry3Aa-AMP fusion crystals were successfully produced and purified (Figure 2B), suggesting that fusion to Cry3Aa aids in abrogating melittin’s toxicity to the host bacteria. Since minimal cleaved melittin (MW ∼ 2 kDa) or promelittin (MW ∼ 2 kDa) bands were observed, we undertook the assumption of a ratio of 1:1 Cry3Aa to AMP molecules in the Cry3Aa-AMP fusion crystal for all subsequent experiments.

### 2.3 Antileishmanial Activity of Cry3Aa-AMP Fusion Crystals

The antiamastigote activities of the Cry3Aa-AMP fusion crystals and their corresponding peptides were evaluated on peritoneal elicited macrophages (PEMs) infected with *L. donovani* LU3 and *L. amazonensis* LV78 over a 72 h incubation period. The native melittin peptide, whose antipromastigote activity had previously been verified against *L. donovani* (Figure S1) and *L. amazonensis* promastigotes (Figure 1A) was set up in parallel to ascertain the impact on melittin’s antileishmanial activity when fused to Cry3Aa. At 0.8 μM concentration, the free melittin and promelittin peptides were ineffective in killing the *Leishmania* amastigotes as there was no meaningful reduction in the *L. donovani* LU3 or *L. amazonensis* LV78 burdens of the infected macrophages compared with no treatment controls (Figure 2C–F). In contrast, fusion to the Cry3Aa crystals appeared to enhance the AMP’s antileishmanial potency toward *L. donovani* LU3 amastigotes as the Cry3Aa-PMLT and Cry3Aa-MLT significantly outperformed their peptide counterparts, which we attributed to the ability of the Cry3Aa crystal framework protecting melittin against proteolytic degradation in the media and enhancing its delivery to the lysosomes of the macrophages where the *Leishmania* reside.^22,23,25^

Meanwhile, it was observed that the LPETG-containing Cry3Aa fusion variants exhibited significantly more effective killing against *L. donovani* LU3, achieving 89% killing for Cry3Aa-mPMLT and 77% killing for Cry3Aa-mMLT, corresponding to 2–3-fold improvement over Cry3Aa-PMLT and Cry3Aa-MLT, respectively (Figures 2C,E and S3). Intriguingly, against *L. amazonensis* LV78, only the LEPTG-containing Cry3Aa-mPMLT and Cry3Aa-mMLT constructs were effective against the parasites—reducing the burden by 63% and 53%, respectively (Figures 2D,F and Figure S3). These results indicated that the LEPTG sequence sandwiched between the pro region and melittin aided in enhancing the antileishmanial activity of the AMPs. As mentioned previously, there has been no report on melittin being effective against *L. amazonensis*, although other studies had reported on the efficacy of melittin against other strains of *Leishmania* responsible for cutaneous leishmaniasis like *L. infantum* promastigotes and amastigotes.^32^ This is the first study demonstrating the effectivity of melittin against LV78 parasites enabled by its fusion to the Cry3Aa crystal platform.

Encouraged by the improved leishmanial killing exhibited by these constructs compared with the free melittin peptide, we moved to study the antileishmanial activities of the two Cry-AMP constructs over a range of concentrations to ascertain their IC_50_’s. The IC_50_ values of Cry3Aa-mPMLT and Cry3Aa-mMLT for *L. donovani* LU3 at 0.3 μM and 0.5 μM, respectively, and *L. amazonensis* at 0.6 μM and 0.8 μM, respectively, were significantly lower than those of the free melittin peptide, which exhibited minimal killing even at 1 μM—the highest concentration that could be tested without concomitant killing of the infected PEMs (Table 1 and Figure S4A,B). Given that the constructs Cry3Aa-mPMLT and Cry3A-mMLT both exhibited much more potent leishmanicidal effects and successfully eliminated intracellular amastigotes of *L. donovani* LU3 and *L. amazonensis* LV78, we opted to focus on these two Cry3Aa fusion variants for further studies.

**Table 1:** IC_50_ of the Melittin Peptide and Melittin-Containing Cry3Aa Fusion Crystals on *L. amazonensis* and *L. donovani* Amastigotes

	amastigotes IC_50_			therapeutic index	
	LU3	LV78	PEMs CC_50_	LU3	LV78
melittin	>1 μM	>1 μM	1.4 μM	<1	<1
Cry3Aa-mPMLT	0.3 μM	0.6 μM	4.1 μM	14	7
Cry3Aa-mMLT	0.5 μM	0.8 μM	3.8 μM	8	5

### 2.4 Reduced Hemolytic Activity and Cellular Toxicity of Cry3Aa-AMP Fusion Crystals

**Figure 3:**
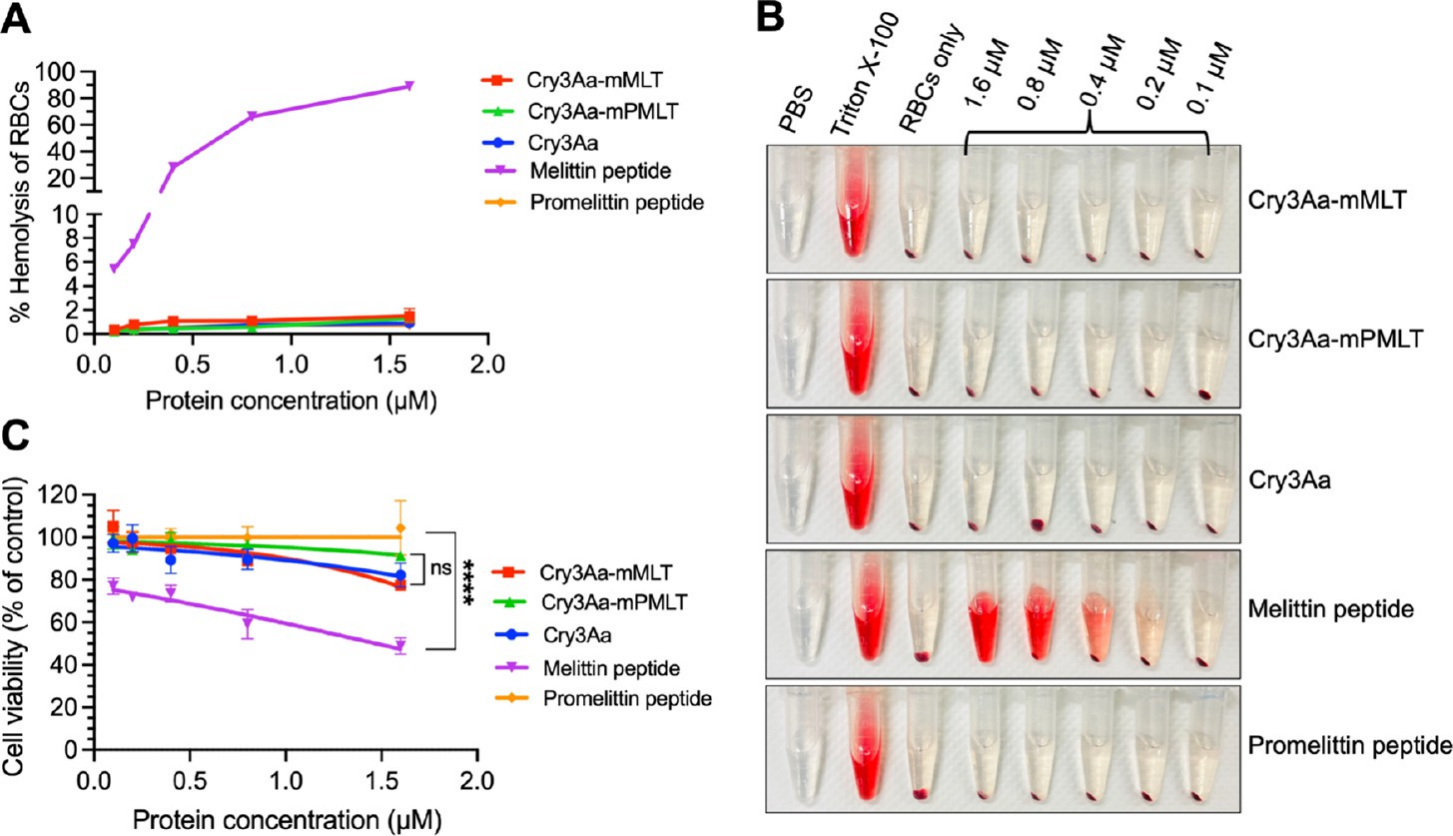
Hemolytic activity and cytotoxicity of Cry3Aa-AMP fusion crystals. (A,B) RBCs were treated with different concentrations (0.1–1.6 μM) of Cry3Aa-mMLT and Cry3Aa-mPMLT fusion crystals and their corresponding free peptide counterparts for 4 h at 37 °C. (A) Note that the free melittin peptides readily lysed the RBCs, while no RBC lysis was observed for the Cry3Aa-mMLT fusion crystals. (B) Release of hemoglobin after RBC lysis could be easily observed due to the color of the supernatant turning red. Triton X-100 was used as a positive control, while PBS solvent was employed as a negative control. (C) Percentage of viable PEMs after treatment with Cry3Aa control, fusion crystals (Cry3Aa-mPMLT and Cry3Aa-mMLT), and their corresponding peptides is shown. ****P < 0.0001. ns, not significant.

Having shown that Cry3Aa-mPMLT and Cry3Aa-mMLT were effective in killing the *Leishmania* parasites, our next priority was then to investigate their effect on the toxicity to mammalian cells. Our hypothesis was that the fusion of melittin to Cry3Aa protein crystals might aid in reducing melittin’s high hemolytic activity and lethality against host cells. Toward this end, mouse red blood cells (RBCs) were incubated with different concentrations of Cry3Aa-mPMLT and Cry3Aa-mMLT fusion crystals as well as their free peptide counterparts for 4 h to evaluate their hemolytic activity. As expected, neither the promelittin peptide nor the Cry3Aa-mPMLT fusion crystals was hemolytic to the RBCs at the concentration tested (Figure 3A,B). On the other hand, the melittin peptide, known for its high hemolytic activity, induced hemolysis at the low concentration of 0.1 μM and 100% hemolysis at 1.6 μM. This was in contrast to the minimal lysis observed for its fusion crystal counterpart, Cry3Aa-mMLT, which exhibited no hemolytic activity for all concentrations tested (Figure 3B). Thus, the fusion of melittin to Cry3Aa crystals significantly suppressed its hemolytic activity.

It has been reported that free melittin peptide is cytotoxic to RAW 264.7 macrophages cells.^33^ The in vitro cytotoxicity of the fusion crystals on PEMs was therefore investigated. Consistent with the findings of the hemolytic investigations, the free melittin peptide was cytotoxic to PEMs and readily killed ∼30% of the cells even at the low concentration of 0.4 μM, whereas the Cry3Aa-mMLT was noticeably less cytotoxic, as evidenced by the much higher number of viable cells at every tested concentration (Figure 3C). Furthermore, the higher CC_50_ values at 4.1 μM for Cry3Aa-mPMLT and 3.8 μM for Cry3Aa-mMLT (Table 1, Figure S4C) showed that fusion of melittin to Cry3Aa significantly improved its therapeutic index (TI)—with a TI value of >1 for the melittin peptide against both LU3 and LV78 compared with TI’s of >10 and 7, respectively, for Cry3Aa-mPMLT and >8 and 5 for Cry3Aa-mMLT (Table 1). Subsequent biosafety evaluation against other mammalian cell lines also confirmed minimal cytotoxicity at the concentrations effective against the LU3- and LV8-amastigotes (Figure S5). Collectively, these data supported the notion that the Cry3Aa crystal platform can help mitigate the hemolytic activity and cytotoxicity of melittin, presumably due to it being encapsulated inside the crystal. Unsurprisingly, neither the promelittin peptide nor the Cry3Aa-mPMLT fusion was cytotoxic to PEMs, even at the highest concentration tested (Figure 3C).

### 2.5 Targeting of Cry3Aa-AMP Fusion Crystals to the Lysosomes of Macrophages

**Figure 4:**
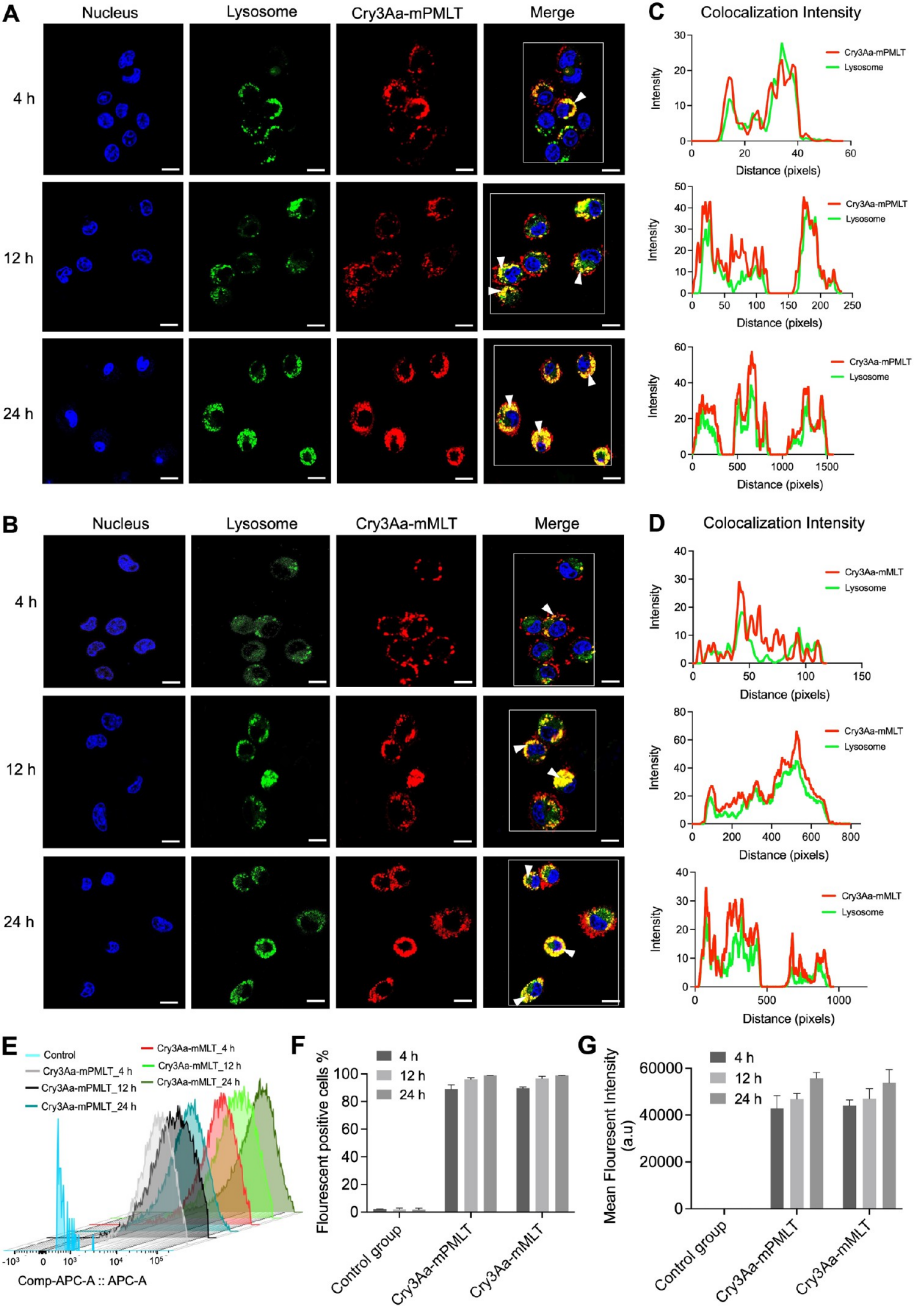
Cellular internalization and colocalization of Cry3Aa-AMP fusion crystals into the lysosomes of macrophages. (A–B) Representative confocal images of RAW 264.7 cells treated with Alexa 647-labeled (red) (A) Cry3Aa-mPMLT fusion crystal and (B) Cry3Aa-mMLT fusion crystal taken at different time points. Efficient internalization of the Cry3Aa-AMP crystals into macrophages could be observed as soon as at the 4 h time point based on the strong red fluorescent signals observed in most cells. Their colocalization with lysosomes is indicated as yellow punctate dots (white arrow) in the merged images. Nuclei of macrophages were stained with Hoechst 33342 (blue), and lysosomes were stained with lysotracker (green). Scale bars: 10 μm. (C,D) Corresponding colocalization intensity profiles of (A) and (B). Macrophages boxed in white in the merged images in (A–B) were used for determining the fluorescence intensity profile generated using ImageJ software. (E–G) Flow cytometric analysis of fusion crystals internalized by macrophages upon incubation with different lengths of time (4, 12, and 24 h). (E) Staggered histograms of the macrophages internalized with labeled fusion crystals and negative controls at different time points. (F) Percentage of macrophages loaded with Alexa 647 labeled fusion crystals and their (G) mean fluorescent intensities (MFI).

*Leishmania* parasites exist as amastigotes in the phagolysosomes of macrophages, which poses a major challenge for most therapeutics as they must overcome the membrane and pH barriers in order to reach the intracellular parasites to act on them.^34^ Previous studies have shown that Cry3Aa crystals can specifically and efficiently be internalized by macrophages but not so by nonphagocytic cells.^24^ To confirm that Cry3Aa-mPMLT and Cry3Aa-mMLT fusion crystals retained this internalization ability, a cellular uptake assay was performed to evaluate the uptake efficiency into macrophages and subsequent localization at different time points over a 24 h period. Confocal micrographs indicated that Alexa-labeled Cry3Aa-mPMLT and Cry3Aa-mMLT fusion crystals were readily uptaken by macrophages, as evidenced by the abundance of the labeled crystals present in most cells after incubation for 4 h and in all cells for 12 h (Figure 4). Their predominant colocalization with the lysotracker-stained lysosomes was also evident from the confocal images (Figure 4A,B) and the corresponding colocalization intensity graphs (Figure 4C,D).

The uptake and lysosomal entrapment were time dependent. At 4 h postincubation, the number of macrophages with internalized Cry3Aa-mPMLT and Cry3Aa-mMLT fusion crystals reached 80% and nearly 100% at 12 h (Figure 4E,F). Meanwhile, the mean fluorescent intensities increased over time, indicating that the individual macrophages were taking up more fusion crystals when incubation time was increased, up to 24 h (Figure 4E,G). The lysosomal entrapment of the internalized fusion crystals increased over time, as revealed by the confocal images showing significantly more yellow punctate fluorescence signals at 12 and 24 h compared with those at 4 h and confirmed by their corresponding intensity profiles displaying fewer red peaks (labeled Cry3Aa-AMP) coincident with the green peaks (lysosome) at the earlier time points (Figure 4C,D). It was noted that the 4 h color profile for the Cry3Aa-mPMLT showed more coincident peaks than the Cry3Aa-mMLT’s.

Although no uptake difference was observed for Cry3Aa-mPMLT and Cry3Aa-mMLT, owing to the more potent antileishmanial activity (Figure 2C–F) and less toxicity to host cells exhibited by Cry3Aa-mPMLT (Figure 3), we decided to focus on Cry3Aa-mPMLT for all the subsequent experiments.

### 2.6 Characterization of Cry3Aa-AMP Fusion Crystals

**Figure 5:**
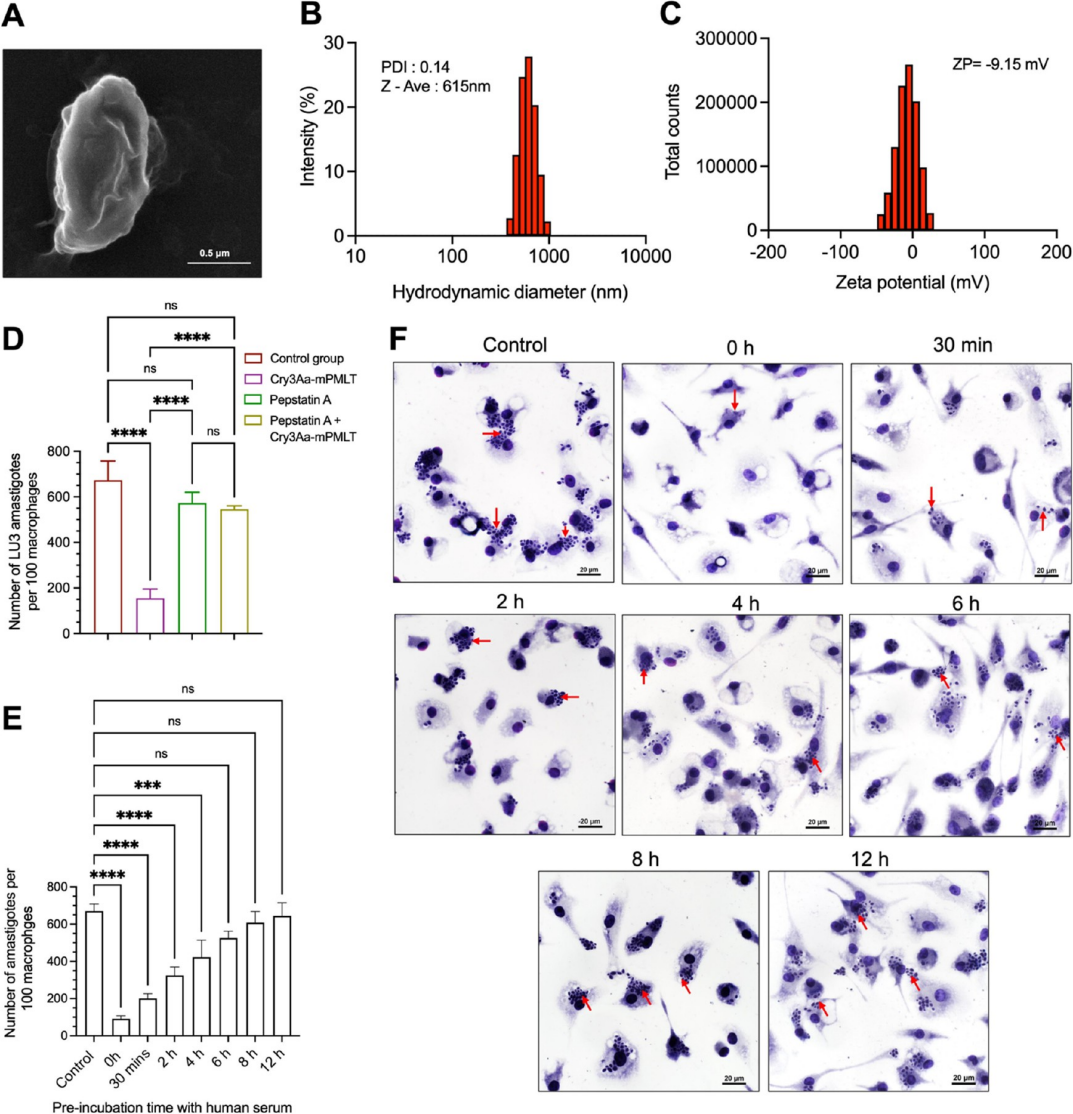
Characterization of Cry3Aa-mPMLT fusion crystals. (A) SEM micrograph and (B) size distribution and (C) zeta potential of the Cry3Aa-mPMLT fusion crystal. **(**D) LU3-infected PEMs were treated with either pepstatin A (20 μg/mL) or Cry3Aa-mPMLT fusion crystals (0.8 μM) or a combination of pepstatin A and Cry3Aa-mPMLT for 72 h at 37 °C. The control groups were untreated parasite-infected-PEMs. At least 100 macrophages were randomly selected across three coverslips of each experimental group for parasite counting. (E–F) Serum stability of Cry3Aa-mPMLT fusion crystals. In vitro antileishmanial activity of Cry3Aa-mPMLT fusion crystals was evaluated by preincubation in serum. (E) Corresponding quantification of the number of *L. amazonensis* LV78 amastigotes in 100 macrophages randomly selected for parasite enumeration. (F) Representative images of LV78-infected PEMs treated with Cry3Aa-mPMLT fusion crystals that were preincubated in human serum for different periods of time (0, 0.5, 2, 4, 6, 8, and 12 h). The control groups were untreated parasite-infected-PEMs. The presence of parasites in macrophages was indicated by the red arrows. ****P < 0.0001 and ***P < 0.001. ns, not significant.

Previous studies have shown that the size, shape, and charge of nanoparticles can affect their cellular internalization and biodistribution.^24,35,36^ Scanning electron microscopy (SEM) revealed that the Cry3Aa-mPMLT fusion crystals were submicrometer-sized particles (Figure 5A) similar to Cry3Aa crystals.^24^ Dynamic light scattering indicated that the crystalline particles were uniform in size, with a mean hydrodynamic diameter of 615 nm (PDI = 0.14) (Figure 5B). The zeta potential of −9.15 mV suggested that the overall charge on the Cry3Aa-mPMLT fusion crystal was slightly negatively charged (Figure 5C). These measurements were comparable to those of the native Cry3Aa crystals, which had demonstrated rapid uptake by RAW 264.7 macrophages in our previous studies.^24^ These observations explain the efficient internalization exhibited by Cry3Aa-mPMLT fusion crystals in the cellular uptake assay.

### 2.7 Role of the LPETG in the Antileishmanial Activity of Cry3Aa-mPMLT Fusion Crystals

Our hypothesis was that insertion of the LPETG sequence before the active melittin sequence would create a preferential cleavage site for the lysosomal cathepsins D/E (Figure S2) that would aid in the release of melittin and thereby enhance its therapeutic efficacy. Consistent with this hypothesis, both Cry3Aa-mPMLT and Cry3Aa-mMLT exhibited significantly more potent antileishmanial activity compared to their corresponding Cry3Aa-PMLT and Cry3Aa-MLT constructs (Figure 2C–F).

To obtain further support, the antileishmanial activity of Cry3Aa-mPMLT was examined in the presence of pepstatin A, a general inhibitor of lysosomal aspartic proteases, including cathepsins D and E. We reasoned that if LPETG was involved in the cleavage of melittin from the fusion crystals mediated by the lysosomal proteases, then the addition of pepstatin A would impede the cleavage and therefore the release of melittin, and in turn, the antileishmanial activity of Cry3Aa-mPMLT. As expected, in the presence of pepstatin A, Cry3Aa-mPMLT was ineffective in killing the amastigotes in the LU3-infected PEMs, as indicated by the comparable parasite burden observed in the untreated control group (Figure 5D). These data support the notion that the inserted LPETG sequence provides a site for the release of melittin from Cry3Aa-mPMLT.

### 2.8 Serum Stability of Cry3Aa-mPMLT Fusion Crystals

Having shown that the Cry3Aa-mPMLT fusion crystals could survive the acidic and proteolytic environment of the phagolysosomes and remain active against the intracellular amastigotes (Figure 2C–F), we next turned our attention to assessing the serum stability of the fusion crystals since the rapid degradation of peptide-based drugs is a major drawback for their systemic therapeutic use, and we hypothesized that the Cry3Aa framework could protect the fused peptide against proteolytic degradation.

The stability of the Cry3Aa-mPMLT fusion crystals against serum degradation was investigated by preincubating the fusion crystals with human serum for different lengths of time and then using them to treat the LV78*-*infected PEMs cultured in 10% FBS-supplemented DMEM for another 72 h to evaluate their residual antileishmanial activity. As shown in Figure 5E,F, the Cry3Aa-mPMLT fusion crystals could reduce the level of intracellular *L. amazonensis* LV78 amastigotes in PEMs by more than 50% after being treated in human serum for 2 h. It is worth mentioning that the length of time the Cry3Aa-mPMLT fusion crystals were exposed to serum proteases was in fact much longer, given that the 72 h treatment was done in 10% FBS-supplemented medium.

### 2.9 In Vivo Toxicity of Cry3Aa-mPMLT Fusion Crystals

**Figure 6:**
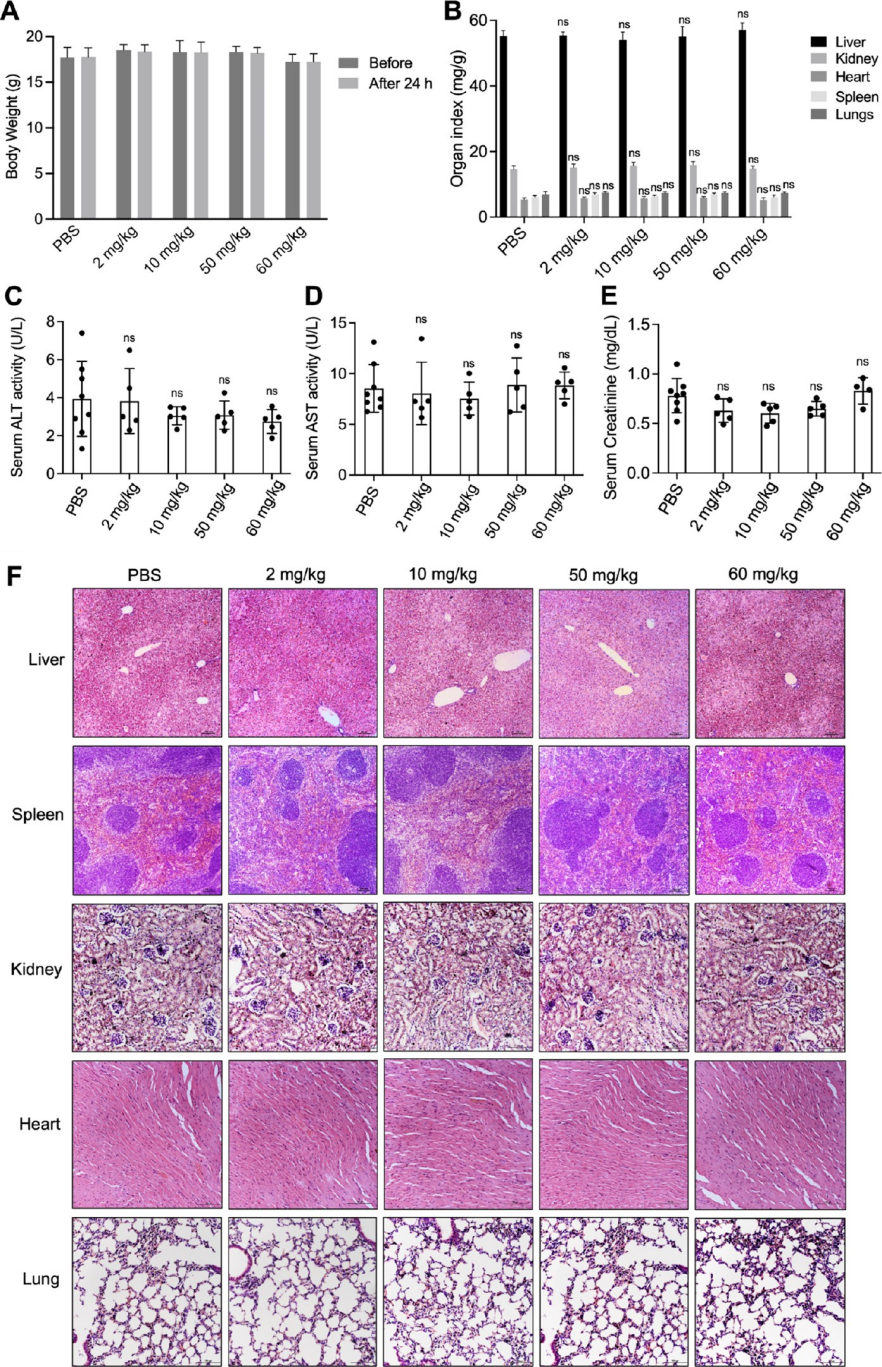
In vivo cytotoxicity of Cry3Aa-mMPLT fusion crystals. (A) Body weight of mice before and after treatment with different dose levels of Cry3Aa-mMPLT fusion crystals (*n* = 5) and the PBS control (*n* = 8). (B) Organ indices of the mice treated with a single dose of Cry3Aa-mPMLT fusion crystals for 24 h. (C–E) Serum levels of the (C) ALT, (D) AST, and (E) creatinine of the blood samples of the fusion crystal-treated mice collected 24 h postadministration. (F) Representative images of the H & E-stained liver, spleen, heart, lungs, and kidney tissues of the differentially treated groups. No obvious injuries or anomalies were observed. ns, not significant.

Having shown that the fusion of melittin to Cry3Aa significantly reduced melittin-induced hemolysis and cytotoxicity in in vitro models, the next step was to determine whether these results could be translated to an in vivo setting. Toward this end, the acute toxicity of Cry3Aa-mPMLT fusion crystals was evaluated in Balb/c mice intravenously injected with a single dose of Cry3Aa-mPMLT fusion crystals (2–60 mg/kg). No body weight change nor organ toxicity was observed for all dose levels tested, as indicated by the comparable weights and organ indices in the treatment group compared with those of PBS control (Figures 6A,B and S6). This was further corroborated by the histological analyses of the stained liver, kidney, lung, heart, and spleen tissues, which showed no abnormalities or substantial damage at any of the tested doses (Figure 6F). Furthermore, biochemical analyses of the serum levels of alanine transaminase (ALT), aspartate transaminase (AST), and creatinine of the treated mice showed no statistical difference from those of the PBS control, even at the highest dose tested (Figure 6C–E). Collectively, these data demonstrated that Cry3A-mPMLT fusion crystals were not toxic to mice for at least up to 60 mg/kg, and this dose could be considered for use in the subsequent in vivo efficacy studies.

### 2.10 In Vivo Efficacy of Cry3Aa-mPMLT Crystal in a Mouse Model of Cutaneous Leishmaniasis

**Figure 7:**
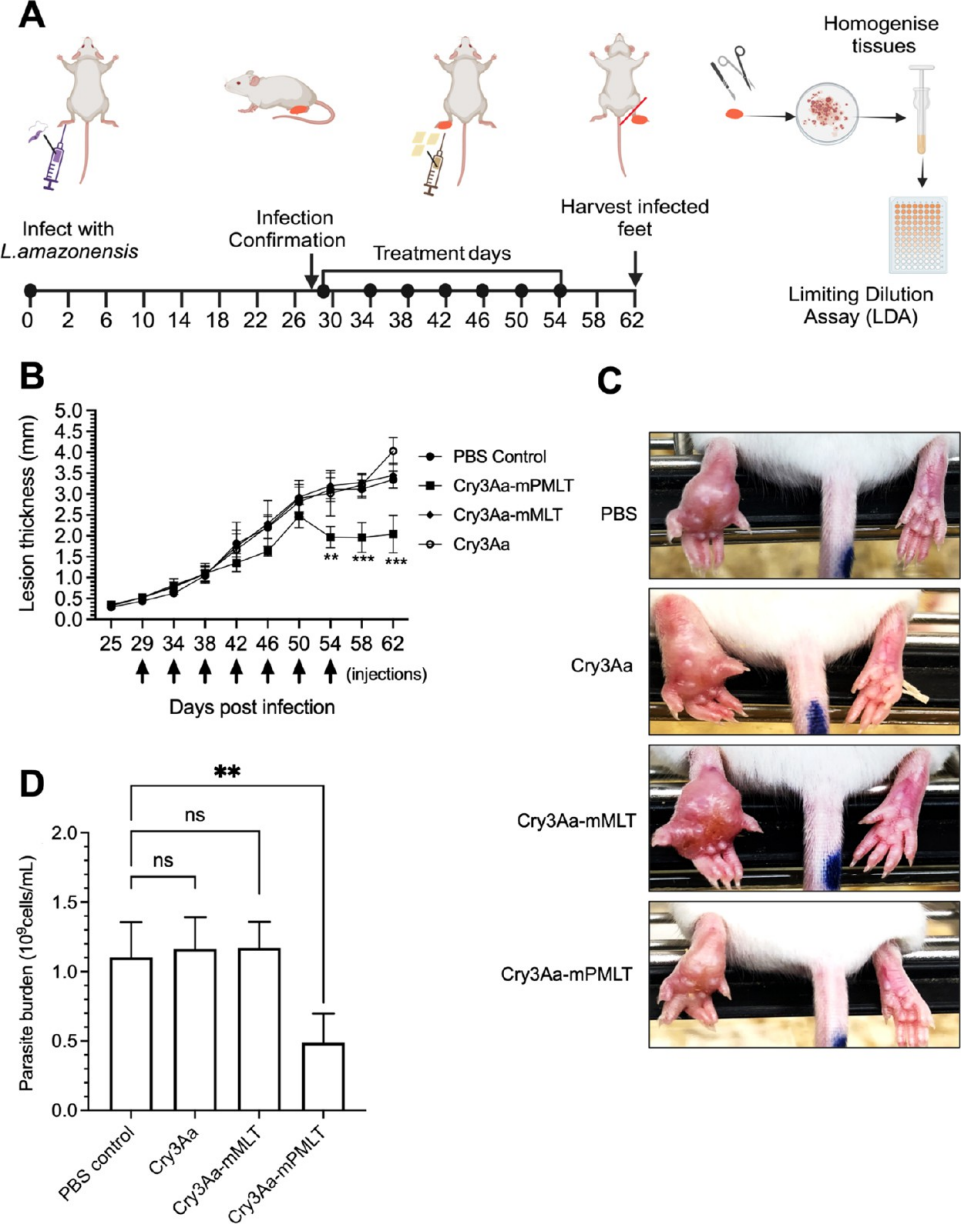
In vivo antileishmanial activity of Cry3Aa-mPMLT and Cry3Aa-mMLT fusion crystals in a mouse model of cutaneous leishmaniasis. (A) Study protocol used for developing footpad *Leishmania* mouse model and treatment schedule. Schematic created with Biorender.com. (B) Thickness of lesions in the left footpads of Balb/c mice infected with *L. amazonensis* LV78 and treated with either PBS, the Cry3Aa crystal group, or the fusion crystal group (Cry3Aa-mMLT and Cry3Aa-mPMLT) over time. Black arrows indicate the days of injections. (C) Representative images showing the morphologic appearance of the differentially treated footpads at day 46 post infection. Significant inflammation and footpad swelling could be observed for the PBS, Cry3Aa, and Cry3Aa-mMLT groups. (D) Parasite burden after the in vitro culturing of the *L. amazonensis* LV8 parasites recovered from the lesions of the differentially treated mice at day 62 post infection. ***P < 0.001 and **P < 0.01. ns, not significant.

The in vivo efficacy of Cry3Aa-mPMLT fusion crystals was first investigated in a murine model of CL in which the left hind footpads were subcutaneously injected with *L. amazonensis* LV78, followed by intralesion treatment over a 4-week period with vehicle controls or Cry3Aa fusions for a total of 7 injections after infection confirmation (Figure 7A). Since Cry3Aa-mMLT had been shown to be effective in killing the amastigotes in PEMs in the in vitro antiamastigote studies (Figure 2C–F), it was also included for parallel comparison with Cry3Aa-mPMLT. The kinetics of lesion development and the lesion morphologic appearance were monitored throughout treatment for disease progression, and the treatment efficacy was determined by measuring the number of amastigotes recovered from the excised footpad lesions after in vitro culturing at the end of the treatment period. No difference in terms of lesion growth kinetics and morphologic appearance was observed for the entire treatment period between the untreated mice in the PBS group and the treated mice in the Cry3Aa control and Cry3Aa-mMLT groups. In contrast, intralesion treatment of the footpad with Cry3Aa-mPMLT effectively inhibited lesion growth following the third administration and led to a significant reduction in lesion thickness at the end of the treatment (Figure 7B,C). Furthermore, the in vitro culturing of promastigotes from the footpad lesions produced significantly fewer parasites for the Cry3Aa-mPMLT treated mice than for the other experimental groups, including Cry3Aa-mMLT (Figure 7D), which was ineffective in inhibiting the lesion growth, despite its promising in vitro performance in treating *L. amazonensis* LV78-infected macrophages.

### 2.11 In Vivo Efficacy of Cry3Aa-mPMLT Crystal in a Mouse Model of Visceral Leishmaniasis

**Figure 8:**
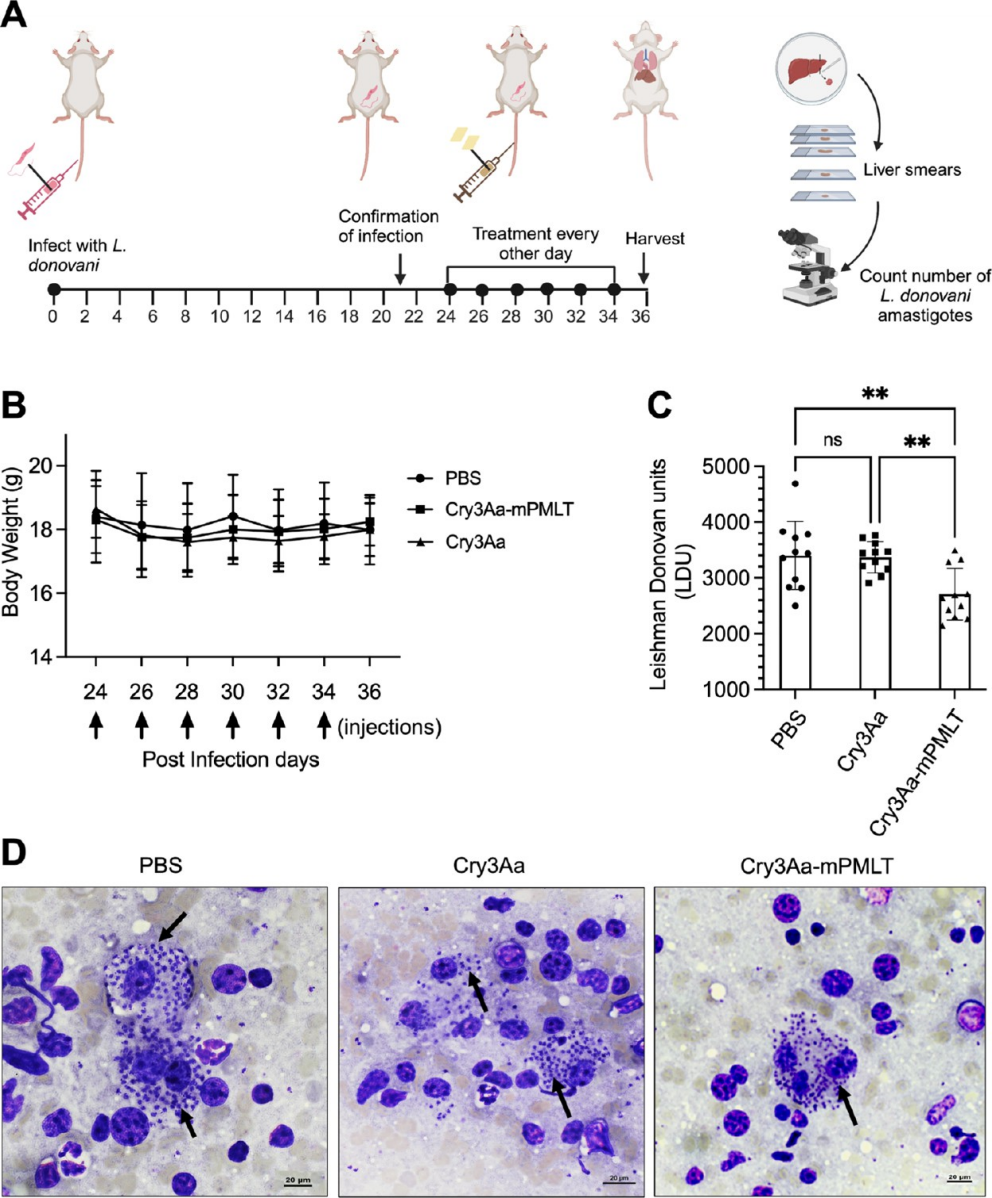
Visceral *Leishmania* mouse model. (A) Schematic of study protocol used for developing visceral *Leishmania* mouse model and treatment schedule created with Biorender.com. (B) Body weight of Balb/c mice infected with *L. donovani* LU3 remained within a normal range during the entire treatment period (*n* = 11). The black arrows represent the days of injection subsequent to the onset of infection. (C) Number of amastigotes per 1000 nucleated cells was counted as Leishman–Donovan units (LDU) in an individual mouse liver smear. (D) LU3-infected liver tissue smear stained with Giemsa. Black arrows indicate amastigotes inside parasitized macrophages. Data were from two independent trials. **P < 0.01. ns, not significant.

Among the three main forms of leishmaniasis, visceral leishmaniasis is the most fatal form, with a > 90% mortality rate if left untreated.^37^ To evaluate the ability of Cry3Aa-mPMLT to treat this form of disease, a mouse model of VL was used. Balb/c mice were intravenously injected with *L. donovani* LU3 promastigotes and, at day 21 post infection, injected intravenously with PBS or 60 mg/kg of either Cry3Aa or Cry3Aa-mPMLT crystals every other day for a total of 6 doses over a 10-day treatment period (Figure 8A). No change in body weight was observed (Figure 8B). 48 h after the last treatment, mice were euthanized, and liver impressions were prepared from the harvested tissues and stained with Giemsa for the microscopic examination and determination of *L. donovani* LU3-amastigote burden. Infected mice treated with the Cry3Aa-mPMLT fusion crystals showed a 28% reduction in parasite load compared to both the PBS and Cry3Aa controls (Figure 8C–D). In addition, an analysis of cytokine mRNA expression in the liver tissues of the differentially treated mice infected with LU3 showed the downregulation of CXCL10 and CXCL2 expression levels in the Cry3Aa-mPMLT treatment group compared to that in the control group (Figure S7).

## 3 Conclusions

This study describes the development of a novel therapeutic, Cry3Aa-mPMLT, for the treatment of cutaneous and visceral leishmaniasis. Key features of these fusion crystals include (1) the direct production of the particle in the *Bt* bacteria already containing the AMP, thereby eliminating the need for separate AMP synthesis and encapsulation; (2) the ability of the Cry3Aa framework to target the antileishmanial melittin peptide to macrophages while concurrently mitigating the AMP’s hemolytic activity and cytotoxicity toward erythrocytes and macrophages and enhancing the in vivo stability of the AMP by protecting it from degradation; and (3) the development of a stimuli-responsive release of the AMP from the therapeutic particle mediated by distinct cathepsin D/E enzymes whose presence are elevated in the acidic environment of infected macrophages. The effectiveness of these fusion constructs for the treatment of *Leishmania*-infected macrophages is demonstrated both in vitro and in vivo using cutaneous and visceral mouse models. It is worth noting that despite the inherent toxicity of melittin, this system was found to be biocompatible and showed no significant cytotoxic effects on either mammalian cells or mice. Thus, we postulate that this platform could provide a general strategy to rationally produce and deliver a variety of AMPs for the treatment of other distinct intracellular infections.

## 4 Experimental Section

### 4.1 Peptide Design and Expression of Cry3A-AMP Fusion Crystals

All the chemically synthesized peptides used in this study were amidated and produced by Pepmic Co., Ltd. (>95% purity). The amino acid sequence of melittin was conserved in all of the peptides with different modifications being introduced to the pro region of melittin to produce the different variants (Figure 2A). The same peptide sequences were genetically fused to the C-terminus of Cry3Aa to produce the corresponding constructs: Cry3Aa-PMLT, Cry3Aa-mPMLT, Cry3Aa-MLT, and Cry3Aa-mMLT, as described below.

Briefly, the synthesized gene encoding wild-type promelittin and modified promelittin optimized for expression in *Bacillus thuringiens*is (*Bt*) (Integrated DNA Technologies) and the *cry3Aa* gene were inserted into the *Xhol* and *Kpn*I sites of the pHT315 vector using Gibson Assembly Master Mix (New England Biolabs). The resultant vectors were used as the template to produce the plasmid constructs Cry3Aa-MLT and Cry3Aa-mMLT by subcloning of the relevant PCR amplicons into the pHT315 vector via Gibson Assembly. After confirmation of the DNA integrity by sequencing (BGI), the plasmids were introduced into *Bt-407-OA* cells by electroporation. The transformed cells were cultured at 25 °C in modified Schaefer’s sporulation medium supplemented with 100 μg/mL erythromycin and 100 μg/mL kanamycin with continuous shaking for 72 h. Harvested cells were extensively washed with ddH_2_0 and 0.5 M NaCl and lysed by overnight incubation with 1 mg/mL lysozyme, followed by sonication. Purified Cry3Aa fusion crystals were obtained by sucrose gradient centrifugation, and their purity was checked under a microscope and verified by SDS-PAGE analysis.

### 4.2 Animal Study

All animal studies were conducted following the protocol approved by the Animal Experimentation Ethics Committee of the Chinese University of Hong Kong (CUHK) and the Department of Health, the Government of the HKSAR under the Animals (Control of Experiments) Ordinance, Chapter 340 (22–1159 in DH/HT&A/8/2/1 Pt. 41).

Female Balb/c mice were used in this study and housed in a controlled temperature room with a 10:14 h light and dark cycle at the Laboratory Animal Service Centre, CUHK. All the animals were fed with a standard diet and water *ad libitum*. Animal experiments that involved parasites *Leishmania amazonensis* LV78 and *Leishmania donovani* LU3 were conducted in the ABSL2 facility in the Laboratory Animal Service Centre, CUHK.

### 4.3 Parasite and Mammalian Cell Cultures

Promastigotes of *L. donovani* LU3 and *L. amazonensis* LV78 were grown in Schneider’s insect medium supplemented with 4 mM glutamine, 50 μg/mL gentamicin, and 10% (v/v) heat-inactivated FBS at 27 and 25 °C, respectively. Murine leukemic, macrophage RAW 264.7 cell line was gifted by Dr. Daniel Lee (Hong Kong Polytechnic University, HKSAR) and cultured in Dulbecco’s modified Eagle’s medium (DMEM) containing 10% (v/v) heat-inactivated fetal bovine serum (FBS) and 1× penicillin–streptomycin (P/S) at 37 °C in a humidified environment with 5% CO_2_.

### 4.4 Scanning Electron Microscopy

The morphology of the Cry3Aa-mPMLT fusion crystal was examined by scanning electron microscopy. The protein concentration of the crystals was first determined using Bradford. The fusion crystals (0.2 mg/mL) were then dehydrated stepwise with 25, 50, 75, and 95% ethanol for 10 min each. Finally, dehydrated crystals were resuspended in 95% ethanol to a final concentration of 0.2 mg/mL. 1 μL of the suspension was deposited onto a round glass slide and allowed to dry overnight at room temperature. On the next day, the samples were sputter coated with gold prior to electron microscopy. SEM imaging was performed with a SU8000 instrument (Hitachi) at a working distance of 8.1 mm and an accelerating voltage of 30 kV.

### 4.5 Dynamic Light Scattering

The size, shape, and zeta potential distribution of the fusion crystals were determined by a Malvern Nano ZS90 (Malvern instrument, U.K.) at 25 °C. 80 μg/mL of Cry3Aa-mPMLT were resuspended in PBS for zeta potential and 150 μg/mL for dynamic light scattering measurements.

### 4.6 Lysosomal Colocalization

Cry3Aa-mMLT and Cry3Aa-mPMLT were labeled with 0.2 μM Alexa Fluor 647 maleimide (Thermo Fischer) in 50 mM HEPES buffer (pH 7.5) for 1 h at room temperature, with constant shaking. The labeled fusion crystals were washed three times with ddH_2_O to remove the excess dye. 1 ×10^5^ RAW 264.7 cells were seeded on confocal dishes (MatTek) and incubated overnight at 37 °C/5% CO_2_ to allow attachment. The next day, nonadherent cells were removed with 1× PBS and replaced with fresh DMEM medium supplemented with 10% FBS and 1 × P/S (complete media). The Alexa 647-labeled fusion crystals (100 nM) were then added to the cells and allowed to incubate for different lengths of time: 4, 12, and 24 h. At the end of the incubation period, the cells were washed twice with PBS and then stained with 0.2 μg/mL Hoechst 33342 (Life Technologies) and 50 nM lysotracker green before imaging on a Leica SP8 confocal microscope.

### 4.7 Quantification of Cry3Aa-AMP into the Macrophages

The amount of Cry3Aa-mPMLT and Cry3Aa-mMLT fusion crystals internalized by macrophage cells was evaluated using flow cytometry. 1 ×10^5^ RAW 264.7 cells/well were seeded in a 12-well plate (Thermo Fischer). Following overnight incubation at 37 °C/5% CO_2_, nonadherent cells were removed with 1× PBS and replaced with fresh complete DMEM media. 100 nM Alexa 647-labeled fusion crystals were added into the wells and allowed to incubate with RAW 264.7 cells at different time points: 4, 12, and 24 h. At the end of the specific incubation period, adherent RAW 264.7 cells were rinsed with cold PBS, harvested, and then analyzed using a BD FACSVerse flow cytometer.

### 4.8 Antipromastigotes Activity

The antipromastigotes activity was determined following a previously described procedure.^38^*L. donovani* LU3 and *L. amazonensis* LV78 promastigotes in log-phase were seeded in 96-well flat-bottom microtiter plates at 1.5 × 10^5^ parasites/well in a final volume of 100 μL of Schneider’s insect medium. Different concentrations of native melittin peptide ranging from 0.5 μM–40 μM and miltefosine from 0.04 to 100 μM were then added to the parasites and incubated for 72 h at their standard culturing temperatures. The viability of the parasites was determined by using an MTS assay. Absorbance was measured at 490 nm wavelength using a TECAN Infinite M1000 plate reader.

### 4.9 Antimicrobial Resistance Development in *Leishmania* promastigotes

Wild-type *L. amazonensis* LV78 strain was subjected to varying concentrations of melittin peptide and miltefosine drugs and incubated at 25 °C for 72 h to determine the initial IC_50_ (designated as passage 0). Afterward, *L. amazonensis* LV78 promastigotes cultivated in a T-25 flask were exposed to drug pressure (lower than the IC_50_). Drug pressure was increased gradually with each passage over 6 weeks until passage 12. At each passage, 1.5 × 10^5^ parasites/well were subjected to a 96-well plate containing a 2-fold serial dilution of melittin peptide and miltefosine drug. MTS assay was performed following 72 h of incubation at 25 °C in order to investigate any changes in the IC_50_ compared to the (passage 0) wild-type strain of *L. amazonensis* LV78.

### 4.10 Hemolysis Assay

Whole blood was collected from Balb/c mice and centrifuged at 10,000 × *g* for 10 min to separate the red blood cells (RBCs) from the serum. RBCs were washed with 1× PBS five times and resuspended in 1× PBS. An 800 μL solution of the RBCs was treated with different concentrations (0.1–1.6 μM) of fusion crystals of Cry3Aa-mPMLT, Cry3Aa-mMLT, Cry3Aa, and melittin and promelittin peptides for 4 h at 37 °C. Parallel treatment of RBCs with PBS and 0.01% Triton X-100 in PBS served as negative and positive controls, respectively. At the end of the treatment period, 100 μL of the supernatant after centrifugation of the reaction mixture was used to measure the absorbance at 570 nm on a TECAN Infinite M1000 Pro plate reader. The degree of hemolysis was calculated using the following formula
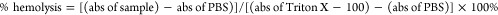


### 4.11 In Vitro Cytotoxicity Studies

Primary mouse peritoneal elicited macrophages (PEMs) were extracted from 5–6-week-old female Balb/c mice, as previously described.^39^ 5 × 10^4^ cells/well were seeded in 100 μL of complete DMEM media in a 96-well flat-bottom microtiter plate and allowed for adherence by incubation at 37 °C/5% CO_2_ for 24 h. On the next day, cells were washed with 1× PBS and treated with graded concentrations (0.1–1.6 μM) of Cry3Aa crystals, Cry3Aa-mMLT, Cry3Aa-mPMLT fusion crystals, and melittin or promelittin peptides resuspended in complete DMEM media for 72 h at 37 °C/5% CO_2_. At the end of the incubation, the cells were washed twice with 1× PBS, and their viability was determined by MTT assay. Absorbance was measured at 565 nm on a TECAN Infinite M1000 pro plate reader. For the derivation of CC_50_, the same procedure was employed, except that the range of concentrations used was 0.05–6.4 μM.

### 4.12 In Vitro Antiamastigotes Activity Assay

PEMs freshly collected from Balb/c mice were seeded at 1 × 10^5^ cell density on a 12 mm round glass slides in a 24-well flat-bottom plate containing 500 μL DMEM complete media and incubated at 37 °C/5% CO_2_ overnight. Cells were then washed with unsupplemented DMEM medium to remove nonadherent cells and RBCs. *L. amazonensis* LV78 at a multiplicity of infection (MOI) of 20 and *L. donovani* LU3 at a MOI of 80 were then added to infect the PEMs overnight at 37 °C with 5% CO_2_, respectively. Miltefosine was set as the positive control. Noninternalized parasites were removed by washing with unsupplemented DMEM medium. Then, 0.8 μM Cry3Aa-AMP fusion crystals, their corresponding peptide counterparts, and Cry3Aa crystals were added to the infected PEMs and incubated at 37 °C for 72 h. At the end of the treatment, cells were washed twice with 1× PBS and fixed with methanol for subsequent staining with the Giemsa. The number of *L. amazonensis* LV78 and *L. donovani* LU3 amastigotes in PEMs was enumerated by examining the fixed cells under the microscope. At least 100 infected PEMs were randomly selected for parasite enumeration. For the determination of IC_50_, the same procedure was employed except for the concentrations used: 0.1–1 μM against *L. amazonensis* LV78 and 0.2–1 μM against *L. donovani* LU3 amastigotes.

### 4.13 Pepstatin A Inhibitor Assay

PEMs infected with *L. donovani* LU3 seeded on glass coverslips were treated with pepstatin A inhibitor (20 μg/mL) or Cry3Aa-mPMLT or a combination of pepstatin A (20 μg/mL) and 0.8 μM Cry3Aa-mPMLT for 72 h at 37 °C/5% CO_2_. At the end of the treatment, cells were rinsed with 1× PBS and fixed with methanol. The fixed cells were then stained with Giemsa for the subsequent enumeration of the number of *L. donovani* LU3 amastigotes in the PEMs. Experiments were performed in triplicate, and at least 100 randomly selected PEMs from each glass slip were examined for parasite enumeration.

### 4.14 Serum Stability of Cry3Aa-mPMLT Fusion Crystals

0.8 μM Cry3Aa-mPMLT fusion crystals were pretreated in serum-free DMEM spiked with 25% (v/v) human serum for different lengths of time (0.5, 2, 4, 6, 8, and 12 h) at 37 °C with gentle shaking. At the end of the indicated incubation period, the treated samples were centrifuged, and the supernatant was removed. The pretreated pellet was washed twice with ddH_2_O and resuspended in complete DMEM medium and then added to LV78-infected PEMs seeded on a glass coverslip. Following a 72 h treatment, the treated PEMs were rinsed twice with 1× PBS, followed by fixation with methanol. The fixed cells were then stained with Giemsa, and the number of amastigotes in the PEMs was enumerated. Experiments were performed in triplicate, and at least 100 randomly selected PEMs from each of the three glass slips were examined for parasite enumeration.

### 4.15 In Vivo Cytotoxicity Studies

7–8-week-old healthy female Balb/c mice were randomized into groups of five and intravenously injected with a single dose of either PBS control (*n* = 8) or different concentrations of Cry3Aa-mPMLT crystals (2, 10, 50, and 60 mg/kg) (*n* = 5) to investigate the *in vivo* acute cytotoxicity of Cry3Aa-mPMLT crystals. Mice were kept in observation for 24 h. At the end of the observation period, mice were sacrificed, and their organs were collected, weighed, and processed for hematoxylin and eosin (H&E) staining for subsequent histological studies. Blood samples were collected at the end of the treatment period, and the serum levels of aspartate aminotransferase (AST), alanine aminotransferase (ALT), and creatinine were determined using Stanbio AST/GOT, Stanbio ALT/GPT, and Stanbio Direct Creatinine LiquiColor, respectively.

### 4.16 RNA Extraction and Real-Time PCR

The mouse liver tissues harvested from the visceral leishmaniasis mouse studies were minced and homogenized in TRIzol (Invitrogen). The homogenates were centrifuged, and the supernatant was used to generate the corresponding cDNA using the iScriptTM advanced cDNA synthesis kit (Bio-Rad). The resultant cDNAs were used for the analysis of the mRNA expression on cytokine and ICAM using the SsoAdvancedTM universal SYBR green supermix (Bio-Rad) on a RT-PCR system (Bio-Rad CFX96). The quantification of mRNA expression levels was conducted by normalizing the protein to GAPDH and utilizing the 2-ΔΔCT method.

### 4.17 Nitric Oxide Detection

20 mg of the liver tissues harvested from the visceral leishmaniasis mouse studies were minced and homogenized in a modified RIPA buffer without SDS and then placed on ice for 10 min. The homogenates were centrifuged at 12,000 × g for 10 min, and the supernatants were transferred to fresh tubes. The samples, together with a 1 mM NaNO_3_ standard, were diluted using ddH_2_O and added to a 96-well plate. Griess reagent prepared following the manufacturer’s instruction (Invitrogen) was then added to the samples in the 96-well plate and incubated for 30 min at room temperature. The absorbance was measured at 548 nm by using a TECAN microplate reader.

### 4.18 Footpad Leishmaniasis Mouse Model

To establish the mouse model of cutaneous leishmaniasis (CL), 3–4-week-old female Balb/c mice were subcutaneously injected with 1 × 10^7^ promastigotes of *L. amazonensis* LV78 in their left hind footpads. The thickness of the footpad lesion was monitored twice a week using a dial caliper. When the lesion thickness reached ∼0.5 mm (day 28 post infection), mice were randomized into the following control and treatment groups (*n* = 5): PBS control, Cry3Aa crystal control, Cry3Aa-mMLT fusion crystal, and Cry3Aa-mPMLT fusion crystal. 50 μL of PBS, Cry3Aa, Cry3Aa-mMLT, or Cry3Aa-mPMLT fusion crystal (0.5 mg/kg for the first dose and 20 mg/kg thereafter) were injected intralesionally and then after an initial 5-day gap every 4 days thereafter for a total of 7 doses over a 4-week period. The lesion was measured prior to each injection, and the lesion thickness was determined by subtracting the measurement from the footpad thickness of the contralateral noninfected foot. Mice were sacrificed 8 days after the last injection, and the left footpads of the mice were removed, cut into tiny pieces, and homogenized in 5 mL of Schneider’s Insect medium supplemented with 10% FBS and 50 μg/mL gentamicin for the quantification of parasite burden by limiting dilution assay (LDA). The homogenized cell suspensions were 10-fold serially diluted and aliquoted into 12 wells of a flat-bottomed 96-well plate. Twelve replicates were prepared for each dilution. The plates were incubated at 25 °C for 2 weeks, and the number of *L. amazonensis* LV78 promastigotes-positive wells was determined using an inverted microscope. The final titer was determined as the dilution at which no parasite was observed in at least one well. The quantification of the parasite burden was achieved by employing the reciprocal of positive titers.

### 4.19 Visceral Leishmaniasis Mouse Model

To establish the murine model of visceral leishmaniasis, 3–4-week-old female Balb/c mice were infected with 5 × 10^7^ promastigotes of *L. donovani* LU3 resuspended in 100 μL of antibiotic-free DMEM (supplemented with FBS) by tail vein injection. The body weight of the infected mice was monitored every other day. After 21 days, two infected mice were euthanized to confirm the infection. In brief, the liver was harvested, and their smears were prepared following standard procedure^40^ and stained with Giemsa to check for the presence of amastigotes.

Once the infection was confirmed, the LU3-infected mice were randomized into the following groups: PBS control group (*n* = 11), Cry3Aa control group (*n* = 11), and Cry3Aa-mPMLT (*n* = 11). Mice were intravenously injected with 100 μL of PBS or 60 mg/kg of either Cry3Aa crystals or Cry3Aa-mPMLT fusion crystals resuspended in 100 μL of PBS every other day for a total of 6 doses. At the end of the treatment period, mice were sacrificed, and their livers were removed. Their organs were weighed, their tissues processed, and liver smear impressions were prepared. The Giemsa-stained liver smears were examined under a microscope to determine the number of amastigotes in tissues. Leishman–Donovan Units (LDU) were obtained by multiplying the number of LU3 amastigotes per 1000 host nuclei by the liver weight (g) of the respective mouse.

### 4.20 Statistical Analysis

Data are expressed as the mean ± standard deviation. Most of the in vitro studies were performed in triplicate unless otherwise indicated. Significant outliers are identified and removed by using Tukey’s fences. Statistical differences were determined by unpaired two-tailed Student’s *t* test for comparison between two groups and one way ANOVA for multiple comparison. All analyses were conducted using GraphPad Prism software version 9.0, and *P* values <0.05 were considered significant.

## Supporting Information Available

Results and Figures: antipromastigote activity of melittin peptide and miltefosine against *L. donovani* promastigotes; cleavage analysis of mPMLT and PMLT peptides by the program Procleave cutter; in vitro antiamastigote activities of different Cry3Aa-AMP fusion crystals against *L. amazonensis* and *L. donovani*; antiamastigote activities of melittin peptide and melittin-containing Cry3Aa fusion crystals evaluated on *Leishmania*-infected PEMs; cytotoxicity assays of melittin peptide and melittin-containing fusion crystals on different mammalian cell lines; percentage of the surviving mice treated with different dose levels of Cry3Aa-mMPLT fusion crystals for 24 h; mRNA expression of cytokines and ICAM in the liver tissues of the LU3-infected mice quantitatively analyzed using real-time PCR; and nitric oxide production in the liver tissues evaluated using Griess reagent (PDF)
