# Supplementary material for: A Targeted and Protease-Activated Genetically Encoded Melittin-Containing Particle for the Treatment of Cutaneous and Visceral Leishmaniasis
Source: ACS Appl Mater Interfaces. 2024 Sep 6;16(37):49148–63. doi: 10.1021/acsami.4c10426 (PMC11420870; doi:10.1021/acsami.4c10426)
Supplement: Supplementary file 1 — am4c10426_si_001.pdf [file am4c10426_si_001.pdf]

Supporting Information

A targeted and protease-activated genetically encoded melittin-containing particle for the treatment of cutaneous and visceral leishmaniasis

Madiha Habib[a], Jiale Zheng[a], Chin-Fung Chan[b], Zaofeng Yang[a], Iris L.K. Wong[b], Larry M.C. Chow[b], Marianne M. Lee[a,] and Michael K. Chan[a,*]*

[a] School of Life Sciences and Center of Novel Biomaterials, The Chinese University of Hong Kong, Shatin, Hong Kong SAR, China.

[b] Department of Applied Biology and Chemical Technology and the State Key Laboratory of Chemical Biology and Drug Discovery, The Hong Kong Polytechnic University, Hung Hom, Hong Kong SAR, China

*Corresponding authors: Michael K. Chan and Marianne M. Lee

Email: michaelkchan88@cuhk.edu.hk, mariannemmlee@cuhk.edu.hk

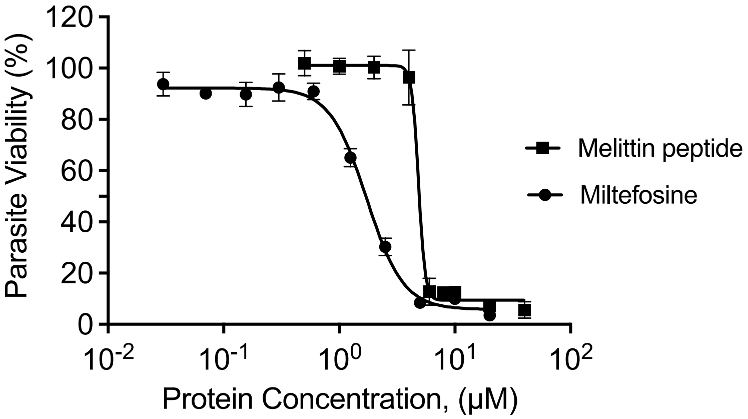

Figure S1. Anti-promastigote activity of melittin peptide and miltefosine against *L. donovani* promastigotes. The IC_{50} values were ~ 2.5 µM and 1.7 µM respectively.

A

mPMLT

Rank	Position	P4-P4' Site	N-fragment	C-fragment	Score	Family
1	41	LPAL†ISWI	4.43 kDa	1.32 kDa	0.425	A01.009
2	44	LISW†IKRK	4.82 kDa	0.93 kDa	0.372	A01.009
3	22	AEAL†PETG	2.25 kDa	3.50 kDa	0.251	A01.009
4	28	TGIG†AVLK	3.04 kDa	2.71 kDa	0.223	A01.009
5	31	GAVL†KVLT	3.32 kDa	2.43 kDa	0.181	A01.009
6	34	LKVL†TTGL	3.66 kDa	2.09 kDa	0.145	A01.009
7	38	TTGL†PALI	4.15 kDa	1.60 kDa	0.088	A01.009
8	26	PETG†IGAV	2.76 kDa	3.00 kDa	0.085	A01.009
9	40	GLPA†LISW	4.32 kDa	1.43 kDa	0.052	A01.009
10	20	ADAE†ALPE	2.07 kDa	3.68 kDa	0.041	A01.009
11	43	ALIS†WIKR	4.63 kDa	1.12 kDa	0.039	A01.009
12	9	EPEP†APEP	0.96 kDa	4.79 kDa	0.038	A01.009
13	47	WIKR†KRQQ	5.22 kDa	0.54 kDa	0.034	A01.009
14	33	VLKV†LTTG	3.55 kDa	2.20 kDa	0.034	A01.009
15	19	EADA†EALP	1.94 kDa	3.81 kDa	0.034	A01.009
16	17	EAEA†DAEA	1.76 kDa	4.00 kDa	0.031	A01.009
17	13	APEP†EAEA	1.36 kDa	4.40 kDa	0.029	A01.009
18	32	AVLK†VLTT	3.45 kDa	2.30 kDa	0.027	A01.009
19	18	AEAD†AEAL	1.87 kDa	3.88 kDa	0.026	A01.009
20	29	GIGA†VLKV	3.11 kDa	2.64 kDa	0.024	A01.009
21	30	IGAV†LKVL	3.21 kDa	2.54 kDa	0.020	A01.009
22	46	SWIK†RKRQ	5.06 kDa	0.69 kDa	0.019	A01.009
23	16	PEAE†ADAE	1.68 kDa	4.07 kDa	0.018	A01.009
24	36	VLTT†GLPA	3.87 kDa	1.89 kDa	0.015	A01.009
25	21	DAEA†LPET	2.14 kDa	3.61 kDa	0.013	A01.009
26	15	EPEA†EADA	1.56 kDa	4.20 kDa	0.013	A01.009
27	37	LTTG†LPAL	4.04 kDa	1.71 kDa	0.013	A01.009
28	14	PEPE†AEAD	1.48 kDa	4.27 kDa	0.013	A01.009

PMLT

Rank	Position	P4-P4' Site	N-fragment	C-fragment	Score	Family
1	41	LPAL†ISWI	4.41 kDa	1.32 kDa	0.425	A01.009
2	44	LISW†IKRK	4.79 kDa	0.93 kDa	0.372	A01.009
3	28	AGIG†AVLK	3.01 kDa	2.71 kDa	0.193	A01.009
4	31	GAVL†KVLT	3.30 kDa	2.43 kDa	0.181	A01.009
5	34	LKVL†TTGL	3.64 kDa	2.09 kDa	0.145	A01.009
6	38	TTGL†PALI	4.12 kDa	1.60 kDa	0.088	A01.009
7	18	AEAD†AEAD	1.87 kDa	3.85 kDa	0.063	A01.009
8	26	PEAG†IGAV	2.73 kDa	3.00 kDa	0.060	A01.009
9	40	GLPA†LISW	4.29 kDa	1.43 kDa	0.052	A01.009
10	43	ALIS†WIKR	4.61 kDa	1.12 kDa	0.039	A01.009
11	9	EPEP†APEP	0.96 kDa	4.76 kDa	0.038	A01.009
12	47	WIKR†KRQQ	5.19 kDa	0.54 kDa	0.034	A01.009
13	33	VLKV†LTTG	3.52 kDa	2.20 kDa	0.034	A01.009
14	17	EAEA†DAEA	1.76 kDa	3.97 kDa	0.031	A01.009
15	20	ADAE†ADPE	2.07 kDa	3.65 kDa	0.029	A01.009
16	13	APEP†EAEA	1.36 kDa	4.37 kDa	0.029	A01.009
17	32	AVLK†VLTT	3.42 kDa	2.30 kDa	0.027	A01.009
18	22	AEAD†PEAG	2.26 kDa	3.47 kDa	0.025	A01.009
19	19	EADA†EADP	1.94 kDa	3.78 kDa	0.025	A01.009
20	29	GIGA†VLKV	3.08 kDa	2.64 kDa	0.024	A01.009
21	30	IGAV†LKVL	3.18 kDa	2.54 kDa	0.020	A01.009
22	46	SWIK†RKRQ	5.03 kDa	0.69 kDa	0.019	A01.009
23	16	PEAE†ADAE	1.68 kDa	4.04 kDa	0.018	A01.009
24	24	ADPE†AGIG	2.48 kDa	3.24 kDa	0.018	A01.009
25	36	VLTT†GLPA	3.84 kDa	1.89 kDa	0.015	A01.009
26	15	EPEA†EADA	1.56 kDa	4.17 kDa	0.013	A01.009
27	37	LTTG†LPAL	4.01 kDa	1.71 kDa	0.013	A01.009
28	14	PEPE†AEAD	1.48 kDa	4.24 kDa	0.013	A01.009

B

mPMLT

Rank	Position	P4-P4' Site	N-fragment	C-fragment	Score	Family
1	31	GAVL†KVLT	3.32 kDa	2.43 kDa	0.989	A01.010
2	41	LPAL†ISWI	4.43 kDa	1.32 kDa	0.986	A01.010
3	44	LISW†IKRK	4.82 kDa	0.93 kDa	0.984	A01.010
4	38	TTGL†PALI	4.15 kDa	1.60 kDa	0.983	A01.010
5	22	AEAL†PETG	2.25 kDa	3.50 kDa	0.295	A01.010
6	32	AVLK†VLTT	3.45 kDa	2.30 kDa	0.186	A01.010
7	34	LKVL†TTGL	3.66 kDa	2.09 kDa	0.166	A01.010
8	20	ADAE†ALPE	2.07 kDa	3.68 kDa	0.089	A01.010
9	26	PETG†IGAV	2.76 kDa	3.00 kDa	0.074	A01.010
10	16	PEAE†ADAE	1.68 kDa	4.07 kDa	0.053	A01.010
11	19	EADA†EALP	1.94 kDa	3.81 kDa	0.037	A01.010
12	40	GLPA†LISW	4.32 kDa	1.43 kDa	0.037	A01.010
13	15	EPEA†EADA	1.56 kDa	4.20 kDa	0.022	A01.010
14	33	VLKV†LTTG	3.55 kDa	2.20 kDa	0.022	A01.010
15	43	ALIS†WIKR	4.63 kDa	1.12 kDa	0.020	A01.010
16	13	APEP†EAEA	1.36 kDa	4.40 kDa	0.014	A01.010
17	28	TGIG†AVLK	3.04 kDa	2.71 kDa	0.014	A01.010
18	17	EAEA†DAEA	1.76 kDa	4.00 kDa	0.012	A01.010
19	14	PEPE†AEAD	1.48 kDa	4.27 kDa	0.012	A01.010
20	21	DAEA†LPET	2.14 kDa	3.61 kDa	0.011	A01.010
21	9	EPEP†APEP	0.96 kDa	4.79 kDa	0.010	A01.010
22	24	ALPE†TGIG	2.48 kDa	3.27 kDa	0.010	A01.010
23	29	GIGA†VLKV	3.11 kDa	2.64 kDa	0.009	A01.010
24	30	IGAV†LKVL	3.21 kDa	2.54 kDa	0.009	A01.010
25	18	AEAD†AEAL	1.87 kDa	3.88 kDa	0.008	A01.010
26	27	ETGI†GAVL	2.87 kDa	2.88 kDa	0.005	A01.010
27	8	PEPE†PAPE	0.86 kDa	4.89 kDa	0.005	A01.010
28	35	KVLT†TGLP	3.77 kDa	1.99 kDa	0.005	A01.010

PMLT

Rank	Position	P4-P4' Site	N-fragment	C-fragment	Score	Family
1	31	GAVL†KVLT	3.30 kDa	2.43 kDa	0.989	A01.010
2	41	LPAL†ISWI	4.41 kDa	1.32 kDa	0.986	A01.010
3	44	LISW†IKRK	4.79 kDa	0.93 kDa	0.984	A01.010
4	38	TTGL†PALI	4.12 kDa	1.60 kDa	0.983	A01.010
5	32	AVLK†VLTT	3.42 kDa	2.30 kDa	0.186	A01.010
6	34	LKVL†TTGL	3.64 kDa	2.09 kDa	0.166	A01.010
7	26	PEAG†IGAV	2.73 kDa	3.00 kDa	0.116	A01.010
8	16	PEAE†ADAE	1.68 kDa	4.04 kDa	0.053	A01.010
9	40	GLPA†LISW	4.29 kDa	1.43 kDa	0.037	A01.010
10	19	EADA†EADP	1.94 kDa	3.78 kDa	0.034	A01.010
11	24	ADPE†AGIG	2.48 kDa	3.24 kDa	0.028	A01.010
12	15	EPEA†EADA	1.56 kDa	4.17 kDa	0.022	A01.010
13	33	VLKV†LTTG	3.52 kDa	2.20 kDa	0.022	A01.010
14	43	ALIS†WIKR	4.61 kDa	1.12 kDa	0.020	A01.010
15	13	APEP†EAEA	1.36 kDa	4.37 kDa	0.014	A01.010
16	17	EAEA†DAEA	1.76 kDa	3.97 kDa	0.012	A01.010
17	14	PEPE†AEAD	1.48 kDa	4.24 kDa	0.012	A01.010
18	9	EPEP†APEP	0.96 kDa	4.76 kDa	0.010	A01.010
19	29	GIGA†VLKV	3.08 kDa	2.64 kDa	0.009	A01.010
20	20	ADAE†ADPE	2.07 kDa	3.65 kDa	0.009	A01.010
21	30	IGAV†LKVL	3.18 kDa	2.54 kDa	0.009	A01.010
22	28	AGIG†AVLK	3.01 kDa	2.71 kDa	0.006	A01.010
23	8	PEPE†PAPE	0.86 kDa	4.86 kDa	0.005	A01.010
24	25	DPEA†GIGA	2.55 kDa	3.17 kDa	0.005	A01.010
25	22	AEAD†PEAG	2.26 kDa	3.47 kDa	0.005	A01.010
26	35	KVLT†TGLP	3.74 kDa	1.99 kDa	0.005	A01.010
27	18	AEAD†AEAD	1.87 kDa	3.85 kDa	0.005	A01.010
28	42	PALI†SWIK	4.52 kDa	1.21 kDa	0.004	A01.010

Figure S2. Cleavage analysis by the program Procleave suggests that the enzyme (A) Cathepsin D exhibits preferential cleavage at the 22nd position of the modified mPMLT peptide compared to the unmodified PMLT peptide, which shows a much lower likelihood of cleavage activity at the 22nd position. Similarly, the enzyme (B) Cathepsin E exhibits a high cleavage specificity at position 22 of mPMLT peptide compared with the unmodified PMLT peptide.

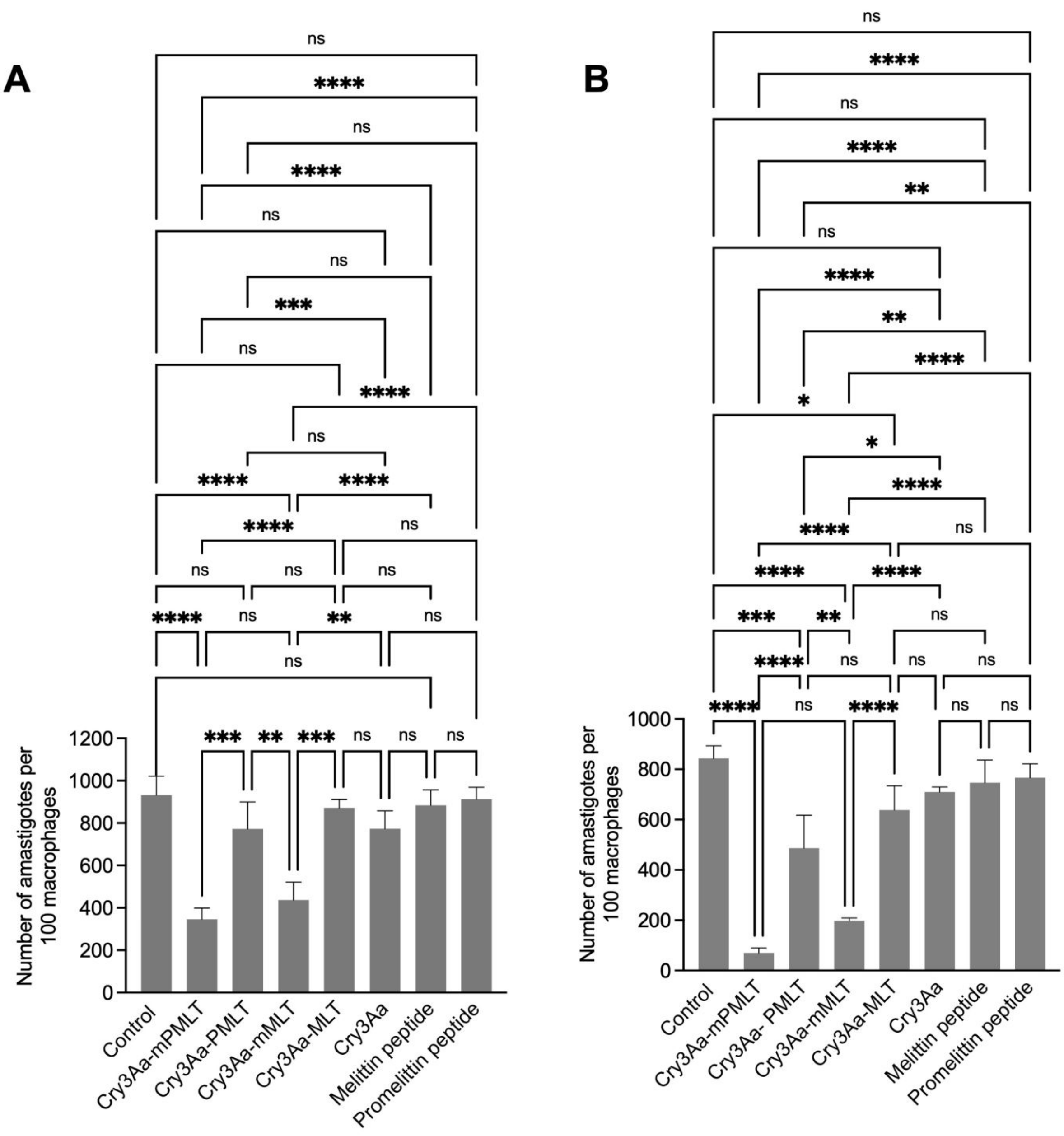

Figure S3. Comparison of in vitro anti-amastigote activities of different Cry3Aa-AMP fusion crystals against (A) *L. amazonensis* LV8 and (B) *L. donovani* LU3. ****$P < 0.0001$, ***$P < 0.001$, **$P < 0.01$, *$P < 0.05$. ns, not significant.

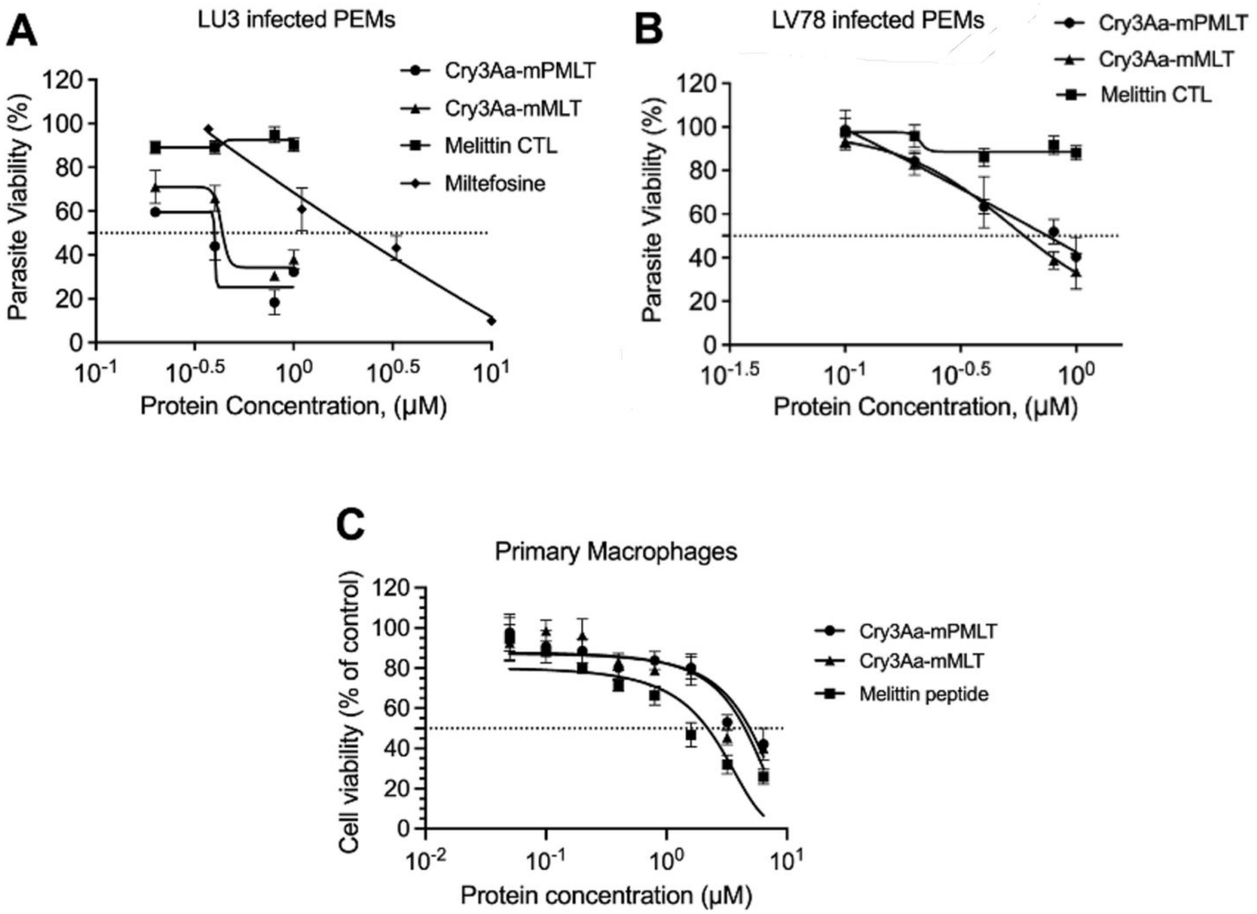

Figure S4. Anti-amastigote activities of melittin peptide and melittin-containing Cry3Aa fusion crystals evaluated on peritoneal elicited macrophages (PEMs) infected with (A) *L. donovani* or (B) *L. amazonensis* over a range of concentrations. Miltefosine was set up in parallel as a positive control. (C) Cytotoxicity assays of melittin peptide and Cry3Aa fusion crystals towards PEMs evaluated over a range of concentrations for the determination of their corresponding CC_{50}'s.

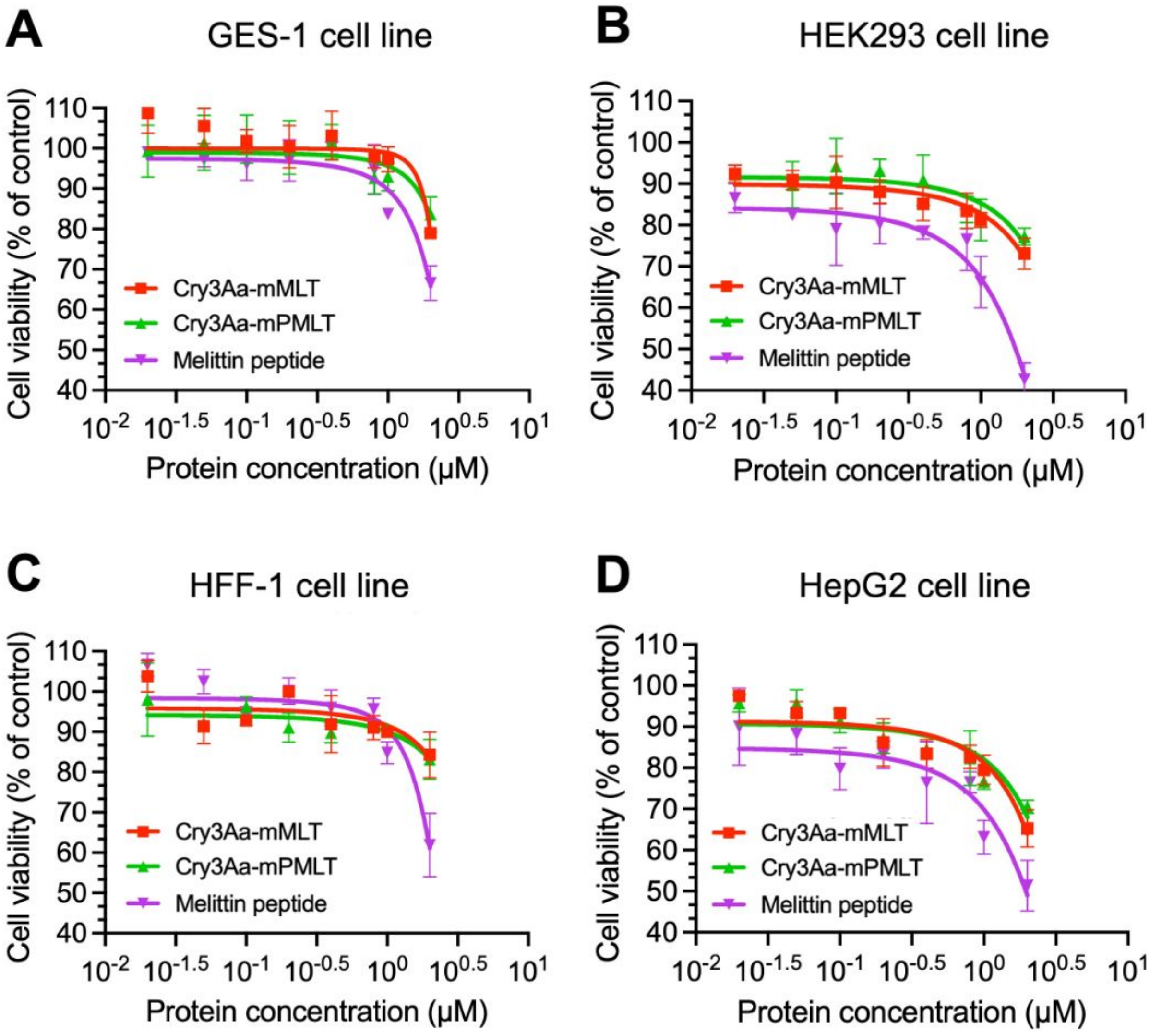

Figure S5. Cytotoxicity assays of melittin peptide and melittin-containing fusion crystals on (A) GES-1, (B) HEK293, (C) HFF-1 and (D) HepG2 cell lines evaluated over a range of concentrations from 20 - 2000 nM.

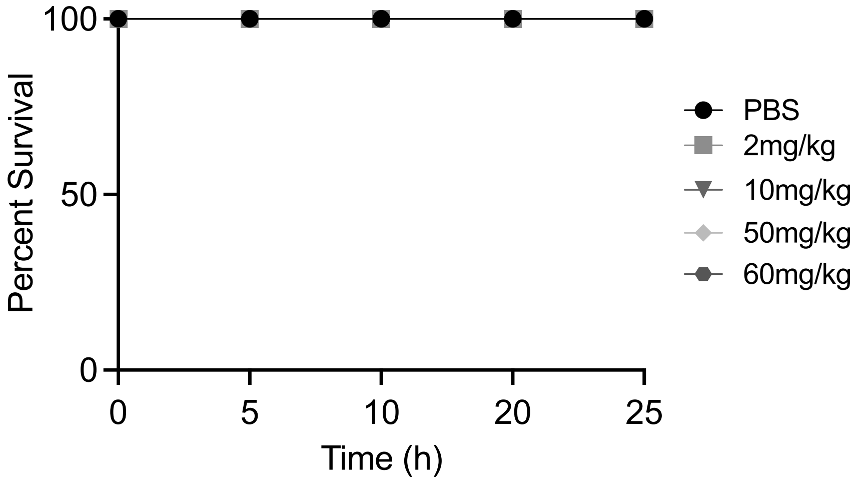

Figure S6. Percentage of the surviving mice treated with different dose levels of Cry3Aa-mPMLT fusion crystals for 24-h.

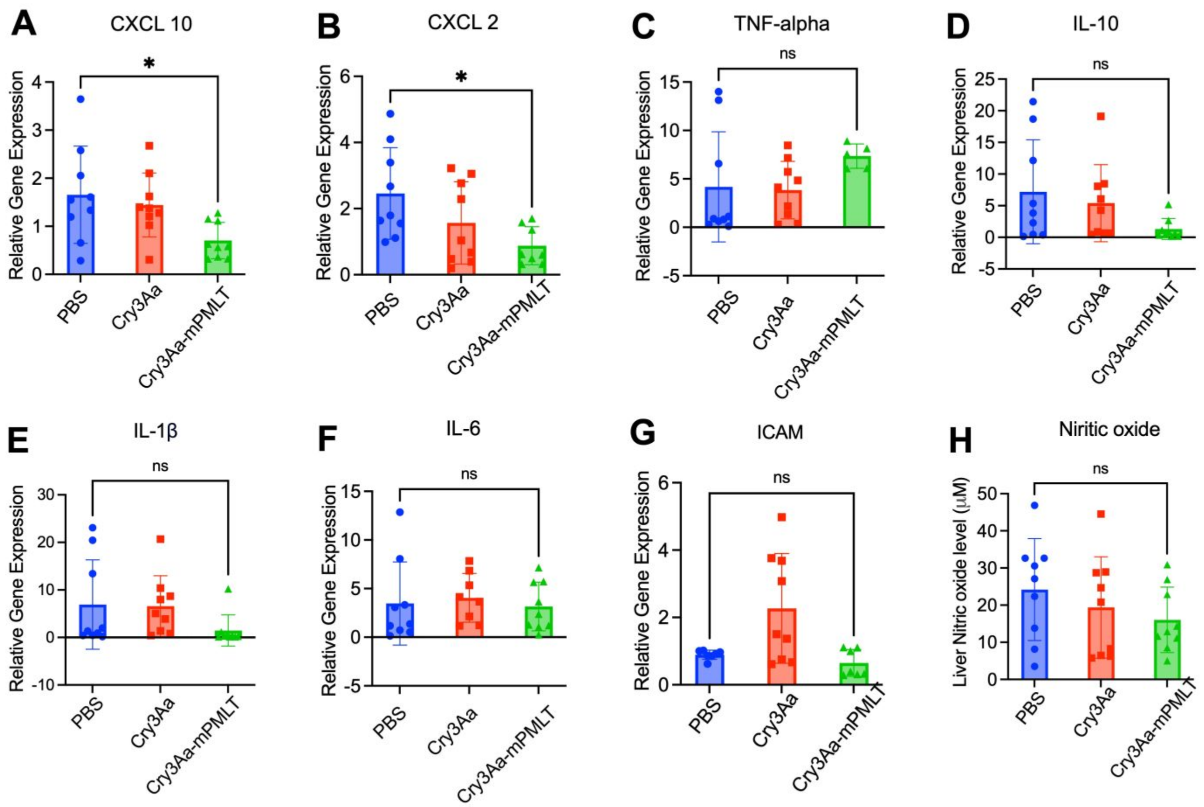

Figure S7. mRNA expression of (A-F) cytokines and (G) ICAM in the liver tissues of the LU3-infected mice quantitatively analyzed using real-time PCR. The mRNA expression was normalized against GAPDH. *$P < 0.05$. ns, not significant. (H) Nitric oxide production in the liver tissues evaluated using Griess reagent.
